# A High-Density Simple Sequence Repeat and Single Nucleotide Polymorphism Genetic Map of the Tetraploid Cotton Genome

**DOI:** 10.1534/g3.111.001552

**Published:** 2012-01-01

**Authors:** John Z. Yu, Russell J. Kohel, David D. Fang, Jaemin Cho, Allen Van Deynze, Mauricio Ulloa, Steven M. Hoffman, Alan E. Pepper, David M. Stelly, Johnie N. Jenkins, Sukumar Saha, Siva P. Kumpatla, Manali R. Shah, William V. Hugie, Richard G. Percy

**Affiliations:** * USDA–ARS, Southern Plains Agricultural Research Center, College Station, Texas 77845; † USDA–ARS, Cotton Fiber Bioscience Research Unit, Southern Regional Research Center, New Orleans, Louisiana 70124; ‡ Seed Biotechnology Center, University of California, Davis, California 95616; § USDA–ARS, WICSRU, Shafter Cotton Research Station, Shafter, California 93263; ** Department of Biology and; †† Department of Soil and Crop Sciences, Texas A&M University, College Station, Texas 77843; ‡‡ USDA–ARS, Genetics and Precision Agriculture Research Unit, Starkville, Mississippi 39762; §§ Dow AgroSciences LLC, Indianapolis, Indiana 46268; *** Monsanto Company, St. Louis, Missouri 63167

**Keywords:** cotton (*Gossypium* spp.) genomes, genetic linkage map, simple sequence repeat (SSR), single nucleotide polymorphism (SNP), recombinant inbred line (RIL) population

## Abstract

Genetic linkage maps play fundamental roles in understanding genome structure, explaining genome formation events during evolution, and discovering the genetic bases of important traits. A high-density cotton (*Gossypium* spp.) genetic map was developed using representative sets of simple sequence repeat (SSR) and the first public set of single nucleotide polymorphism (SNP) markers to genotype 186 recombinant inbred lines (RILs) derived from an interspecific cross between *Gossypium hirsutum* L. (TM-1) and *G. barbadense* L. (3-79). The genetic map comprised 2072 loci (1825 SSRs and 247 SNPs) and covered 3380 centiMorgan (cM) of the cotton genome (AD) with an average marker interval of 1.63 cM. The allotetraploid cotton genome produced equivalent recombination frequencies in its two subgenomes (At and Dt). Of the 2072 loci, 1138 (54.9%) were mapped to 13 At-subgenome chromosomes, covering 1726.8 cM (51.1%), and 934 (45.1%) mapped to 13 Dt-subgenome chromosomes, covering 1653.1 cM (48.9%). The genetically smallest homeologous chromosome pair was Chr. 04 (A04) and 22 (D04), and the largest was Chr. 05 (A05) and 19 (D05). Duplicate loci between and within homeologous chromosomes were identified that facilitate investigations of chromosome translocations. The map augments evidence of reciprocal rearrangement between ancestral forms of Chr. 02 and 03 versus segmental homeologs 14 and 17 as centromeric regions show homeologous between Chr. 02 (A02) and 17 (D02), as well as between Chr. 03 (A03) and 14 (D03). This research represents an important foundation for studies on polyploid cottons, including germplasm characterization, gene discovery, and genome sequence assembly.

Cotton belongs to the *Gossypium* genus, which consists of approximately 45 diploid and 5 allotetraploid species of global distribution (Beasley 1942; Endrizzi *et al.* 1985; Kohel *et al.* 2001; Stewart 1994; Wendel and Cronn 2003). The gametic chromosome number of all diploid species is 13, but significant differences among the genomes in meiotic affinity and relative size led to the recognition of eight genome groups: A through G and K (Beasley 1942; Endrizzi *et al*. 1985; Stewart 1994). Of the approximately 50 *Gossypium* species, four have been domesticated independently: two diploid species, *G. arboreum* L. and *G. herbaceum* L. (n = x = 13) with A_1_ and A_2_ genomes, and two allotetraploid species, *G. hirsutum* L. and *G. barbadense* L. (n = 2x = 26) with (AD)_1_ and (AD)_2_ genomes (Bowers *et al.* 2003; Lee 1984; Percival and Kohel 1990). The allotetraploid cotton species are the products of a presumed single polyploidization event between ancient A-genome and D-genome diploids that occurred approximately 1-2 million years ago (Stelly *et al.* 2005; Wendel and Cronn 2003). Chromosome numbers assigned in allotetraploid cottons are based on pairing relationships in diploid x tetraploid crosses, with chromosomes 1−13 corresponding to the At subgenome and chromosomes 14−26 to the Dt subgenome (Brown 1980).

Cotton species serve as a model system for polyploid plants and plant cell elongation, cell wall and cellulose biosynthesis because they are the only known plants that produce single-celled fibers (Jiang *et al.* 1998; Kim and Triplett 2001). The genes that make cotton valuable function in unique ways, requiring long-term research into the development of molecular tools such as DNA markers and genome maps to translate genomic information into agronomic benefits and to other biological systems.

Cotton researchers have explored genetic mapping with multiple types of DNA markers, including restriction fragment-length polymorphism (RFLP) (Reinisch *et al.* 1994; Rong *et al.* 2004; Shappley *et al.* 1998), amplified fragment-length polymorphism (Lacape *et al.* 2003), random-amplified polymorphic DNA (Kohel *et al.* 2001), and simple sequence repeats (SSRs) (Guo *et al.* 2007; Lacape *et al.* 2009; Park *et al.* 2005; Xiao *et al.* 2009; Yu *et al.* 2011). Although early genetic mapping with hybridization-based markers such as RFLP opened the door to important genomic studies (Jiang *et al.* 1998; Shappley *et al.* 1998), recent genetic mapping with polymerase chain reaction (PCR)-based markers such as SSR have facilitated portable applications among different mapping populations and research programs (Abdurakhmonov *et al.* 2008; Zhang *et al.* 2003). As such, the cotton research community has made efforts to develop many portable markers to overcome the problem of low DNA polymorphism rates among various cultivated cotton breeding programs (http://www.cottonmarker.org/; Blenda *et al.* 2006). To date, approximately 17,000 pairs of SSR primers have been developed from four cotton species (*G. arboreum*, *G. barbadense*, *G. hirsutum*, and *G. raimondii* Ulbrich) and a portion of this number have been surveyed for polymorphism against a 12-genotype panel of six *Gossypium* species (Blenda *et al.* 2006; Yu 2004). As single nucleotide polymorphism (SNP) markers are explored in other plant species (Ganal *et al.* 2009), new research has been initiated to examine nucleotide sequence diversity in *Gossypium* genomes (An *et al.* 2008; Van Deynze *et al.* 2009). These findings are laying the groundwork for developing large numbers of SNP markers in cotton. The growing collection of portable markers in cotton provides a cost-effective tool for genome mapping and gene discovery to understand and improve the cotton plant.

High-resolution mapping in cotton has been conducted with segregating populations that were derived from interspecific crosses between *Gossypium* species because of limited DNA polymorphism within a cotton species. The resulting segregating populations used in major mapping projects often were either F_2_ or BC_1_ progeny (Guo *et al.* 2007; Lacape *et al.* 2003; Rong *et al.* 2004; Yu *et al.* 2011). In addition, these maps relied heavily on a single marker type such as RFLP or SSR markers derived from limited sources. Rong *et al.* (2004) reported the first high-density map in cotton using 57 F_2_ plants derived from an interspecific cross between *G. hirsutum* race “palmeri” and *G. barbadense* acc. “K101.” The majority of markers used in this map were RFLP markers. This map provided one of the first insights into the allotetraploid cotton genome structure and evolution, although the RFLP markers have proven to have limited portability and utility for marker assisted breeding (Ulloa *et al.* 2005).

Guo *et al.* (2007) reported the first comprehensive SSR map by using 138 BC_1_ plants derived from an interspecific cross of *G. hirsutum* TM-1/*G. barbadense* Hai 7124//*G. hirsutum* TM-1. The majority of SSR markers in this map were derived from cotton expressed sequence tag (EST) sequences. Lacape *et al.* (2009) reported a genetic linkage map that consisted of a total of approximately 800 (amplified fragment-length polymorphism, RFLP, and SSR) marker loci via the use of 140 recombinant inbred lines (RILs); derived from an interspecific cross between *G. hirsutum* Guazuncho 2 and *G. barbadense* VH8-4602. Recently, Yu *et al.* (2011) used 141 BC_1_ plants derived from an interspecific cross of *G. hirsutum* Emian 22/*G. barbadense* 3-79//*G. hirsutum* Emian 22. As with Guo *et al.* (2007), this map also contained SSR markers, the majority of which were derived from ESTs. In addition, a whole-genome radiation hybrid population of 93 plants derived from an interspecific cross of *G. barbadense* 3-79/*G. hirsutum* TM-1 was also explored for mapping the cotton genome (Gao *et al.* 2004, 2006).

Here we report the development of a high-density cotton (*Gossypium* spp.) genetic map by using representative sets of SSR markers and the first public set of SNP markers to genotype 186 RILs derived from an interspecific cross between *G. hirsutum* TM-1 and *G. barbadense* 3-79. Both TM-1 and 3-79 are considered genetic standards for their respective species because of breeding and history of genetic/genomic research conducted by the cotton community. These two lines are highly homozygous, and extensive genetic and cytogenetic materials have been developed using them as reference parents, including mutants and hypoaneuploids (Kohel *et al.* 1970; Stelly 1993; Stelly *et al.* 2005). RILs possess several advantages over F_2_ or BC_1_ populations for mapping genes and quantitative trait loci (QTL), and high levels of homozygosity and recombination in the RILs enable replicate studies across different environments by different research groups. This immortal TM-1 × 3-79 RIL population is maintained at USDA-ARS, College Station, Texas, USA, and it is used by the cotton research community for genetic investigations, including QTL mapping studies. In addition, we selected SSR markers derived from different sequence sources (EST, genomic, and BAC clones). These markers were developed by 16 research groups (all 16 sources available to the public at Cotton Marker Database (http://www.cottonmarker.org/). This combined high-density genetic map will facilitate the advancement of many basic and applied genomic studies in cotton.

## Materials and Methods

### Plant materials and DNA extraction

The mapping population was an immortalized set of 186 RILs. At the time of genomic DNA extraction for this study, the average generation was F_7_. These lines were derived from selfing via single-seed descent original individual F_2_ plants from a cross between *G. hirsutum* TM-1 and *G. barbadense* 3-79, two highly homozygous parents (Kohel *et al.* 1970; Niles and Feaster 1984). Factors in selecting TM-1 and 3-79 as parents in creation of a segregating population for genetic mapping are the unique high-quality fiber characteristics of extra long staple cotton 3-79 and the high productivity and modest environmental sensitivity of Upland cotton TM-1 (Kohel *et al.* 2001). The parents (TM-1 and 3-79) and their 186 RIL progeny are maintained as living specimens to produce seed, fiber, and leaf tissue for this mapping effort and other genetic studies.

Interspecific F_1_ hypoaneuploid hybrids for specific chromosomes were used for deficiency mapping by means of loss of heterozygosity. All but one were derived previously by pollinating monosomic and monotelodisomic aneuploids quasi-isogenic to TM-1 with pollen from euploid 3-79, and recovering the respective deficiency among F_1_ progeny. The F_1_ aneuploid monosomic for chromosome 26 was unusual in that the deficiency arose *de novo* in 3-79 pollen, *i.e.* not via transmission from the maternal TM-1−like stock. The general procedures for mapping with cotton monosomic (2n = 51) and monotelodisomic stocks have been described previously (Beasley 1942; Stelly 1993; Stelly *et al.* 2005).

Genomic DNA was extracted from fresh young leaf tissue of individual cotton plants grown in the greenhouse in accordance with the modified CTAB DNA extraction procedure as described by Kohel *et al.* (2001).

### PCR primers and assays

The primer pairs used for PCR were developed by collaborators of the cotton research community (Table 1). Approximately 10,000 pairs of SSR primers from 16 different research projects (http://www.cottonmarker.org*/*) were first analyzed to identify polymorphic markers between TM-1 and 3-79. Nine genomic DNA sources for SSR primer pairs included BNL, CIR, CM, DOW, DPL, GH, JESPR, MUSB, and TMB, and seven EST sources of SSR primer pairs included HAU, MGHES, MUCS, MUSS, NAU, STV, and UCD. While EST SSR primer pairs were developed from *Gossypium* cDNA clones that contain SSR, genomic primer pairs were developed from *Gossypium* random enriched small insert libraries except MUSB and TMB. MUSB was developed from the end sequences of the bacterial artificial chromosome (BAC) clones of *G. hirsutum* acc. Acala Maxxa (Frelichowski Jr. *et al.* 2006). TMB was developed from the BAC clones and/or physical contigs of TM-1 (Guo *et al.* 2008). MUSB and TMB markers facilitate an integration of genetic and physical maps of the allotetraploid cotton genome (Xu *et al.* 2008). The first public SNP set (UC) also was included in this mapping project (Van Deynze *et al.* 2009). SNP primer pairs were largely derived from *G. arboreum* EST unigenes. The actual sequence of the individual primer pairs and source clone for each SSR or SNP marker set can be found at http://www.cottonmarker.org/.

PCR assays for amplifying SSR markers were performed in a cocktail of 10 μL containing 20 ng of DNA, 0.25 μM forward primer, 0.25 μM reverse primer, 0.25 mM dNTPs, 2.5 mM MgCl_2_, and 0.65 unit DNA Taq polymerase. Thirty-five PCR cycles were used to amplify SSR products, using a primer annealing temperature of 55° or 60°. For nonlabeled SSR primers, amplified DNA products were electrophoresed in a 20-cm-long horizontal agarose gel system (Owl Separation Systems, Portsmouth, NH) with 1X TBE (45 mM tris-borate, 1 mM EDTA, pH 8) running buffer and 3.5% Hi-Resolution agarose (*e.g.* Metaphor agarose, Cambrex, East Rutherford, NJ; or SFR agarose, Amresco, Solon, OH). PCR product sizes were estimated by comparison with DNA size standard ladders (E and K Scientific, Santa Clara, CA). For fluorescently labeled primers (forward primer only with 6-FAM, HEX, or NED), amplified DNA products were separated using 36-cm or 50-cm capillary electrophoresis of automated ABI PRISM 3130xl or ABI PRISM 3730 Genetic Analyzer (Applied Biosystems/Life Technology, Foster City, CA). In a separate project, an array for Ilumina (San Diego, CA) Golden Gate assay was designed to analyze 384 SNP markers between TM-1 and 3-79 (Van Deynze *et al.* 2009). Polymorphic SNP markers based on the parental survey were used to genotype the 186 RILs.

### Marker data acquisition and linkage map construction

SSR data collection was performed either manually for gel-based assays or with the GeneMapper 3.7. Among nearly 10,000 pairs of primers that were surveyed, more than 2000 primer pairs that detected the best resolution of polymorphisms between TM-1 and 3-79 were selected to genotype the 186 RILs. These primer pairs included subsets (54 MUCS, 123 MUSB, and 93 MUSS) that were previously used to genotype the same population (Park *et al.* 2005; Frelichowski Jr. *et al.* 2006), and the genotyping data were incorporated into this mapping project.

Genotyping of the RIL population for SSR and SNPs was performed as previously described (Park *et al.* 2005; Frelichowski Jr. *et al.* 2006; Van Deynze *et al.* 2009). SSR markers were generally codominant, but the calling or scoring of the tetraploid cotton alleles at a specific locus required careful examination of gel images or electrographs. Allotetraploid cottons likely had multiple copies of DNA fragments or alleles amplified with a single primer-pair. To distinguish dominant markers from codominant markers, any RIL missing one pair of the parental polymorphic fragments/alleles indicated that alleles were nonallelic or simply an existence of two dominant marker loci after all pairing attempts had failed. A missing data point of a RIL was determined if there was a lack of any signal attributable to failed PCR amplification. Duplicate marker loci were designated by adding a lower-case letter in alphabetical order after the primer name. The raw scores were first inspected for any coding error and segregation distortion before using the data as input for the JoinMap 4.0 program (Van Ooijen 2006) for mapping analysis. Using the JoinMap’s function “identify identical loci,” we identified 47 identical or cosegregating loci (supporting information, Table S2) and removed them in subsequent mapping. The Kosambi mapping function (Kosambi 1944) was selected to convert a recombination frequency to a genetic distance (centiMorgan, or cM), and 40 cM was the threshold to determine linkage between two markers. Linkage groups and marker orders were determined on the basis of likelihood ratio statistic (or LOD) 10 or greater (up to LOD 15). Chromosome assignment was determined by the common markers that were located by authors in previous publications (Frelichowski Jr. *et al.* 2006; Guo *et al.* 2007, 2008; Lacape *et al.* 2003; Liu *et al.* 2000; Park *et al.* 2005; Yu *et al.* 2011) and by use of the subsets of new SSR markers (GH, Table 3) with the cotton hypoaneuploid stocks described previously. SSR loci localized to one of the chromosomes (Chr.) 1 to 13 were assigned to the A-subgenome (At), whereas loci localized to Chr. 14 to 26 were assigned to the D-subgenome (Dt).

## Results

### Parental polymorphisms and genotype frequencies of the mapping population

Approximately 25% of the genomic SSR markers and approximately 15% of the cDNA SSR markers were polymorphic between TM-1 and 3-79. A total of 1601 pairs of polymorphic SSR primers were selected and analyzed for genotyping 186 RILs. Of the 1601 SSR primer pairs that revealed 1895 marker loci, 1344 primer pairs revealed one locus, 234 revealed two loci, and the remaining 23 revealed more than two loci. Among the 1895 SSR marker loci, 1785 were codominant; 43 were dominant loci that received alleles from TM-1, and 67 were dominant loci that received alleles from 3-79. Of these 1895 marker loci, 1825 were mapped (Table 1). The remaining 70 loci were not mapped because of highly skewed segregation (χ^2^ > 8.5) and high levels of missing data. Fifty-five of the unmapped loci were dominant loci. In addition, 247 of the 384 SNP primer pairs were polymorphic between parents and used to genotype the 186 RILs. All 247 SNP markers were codominant and revealed 247 loci (Table S1). Of these, 207 SNP loci were mapped in unique positions, and the remaining 40 SNP loci were identical to other mapped loci (Table S2). In summary, a total of 1848 pairs of SSR and SNP primers were used to genotype the 186 RILs, and 2142 marker loci were scored, of which 2072 marker loci revealed by 1532 pairs of SSR and 247 pairs of SNP primers were mapped (Table 1). Approximately 98% of 2072 total marker loci were mapped in unique positions, with only 47 identical or cosegregating markers including 40 SNP markers (Table S2).

**Table 1:** Primer sources of cotton molecular markers (http://www.cottonmarker.org/)

Marker set	No. Mapped Marker Loci	No. Mapped Primer Pairs
Genomic SSRs		
BNL	304	239
CIR	123	104
CM	32	24
DOW	60	60
DPL	213	200
GH	149	144
JESPR	122	89
MUSB	155	123
TMB	310	266
Subtotal	1468	1249
EST SSRs		
HAU	12	8
MGHES	20	14
MUCS	63	54
MUSS	112	93
NAU	113	90
STV	9	7
UCD	28	17
Subtotal	357	283
SNPs		
UC	247	247
Total	2072	1779

EST, expressed sequence tag; SNP, single nucleotide polymorphism; SSR, simple sequence repeat.

**Figure 1:**
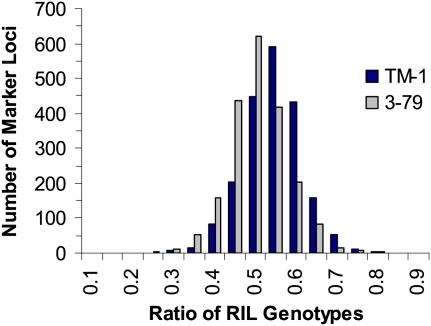
Distribution of the TM-1 and 3-79 allele frequencies in the RIL mapping population (χ^2^ = 768 and *P* < 0.0001).

This RIL population displayed a greater-than-expected level of residual heterozygosity, *i.e.* 4.2% instead of the expected 1.6% for an F_7_ population derived by single-seed descent. Residual heterozygosity in individual lines ranged from 0.8% to 19.9%. Among the 2032 codominant SSR and SNP loci, the average residual heterozygosity for individual markers was 4.2%, ranging from 0% to 66.7% with SSR marker STV129 demonstrating the greatest heterozygosity. Markers that detected more than 20% residual heterozygosity of the RIL population were usually difficult to map because determining linkage of these markers conflicted with more than one marker. Analysis of the genotyping data revealed a statistically significant preference of TM-1 alleles to 3-79 alleles (*χ^2^* = 768; Figure 1). Overall, the allele frequencies of TM-1 and 3-79 were 52.3% and 47.7%, respectively.

### Genetic linkage maps of the allotetraploid cotton

**Figure 2:**
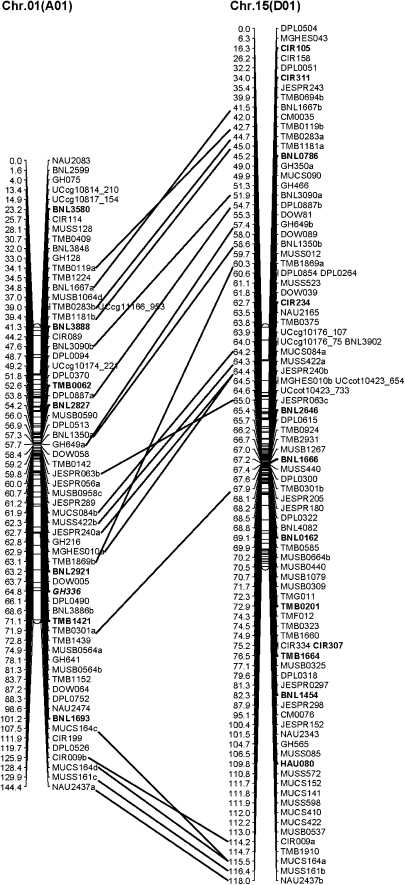

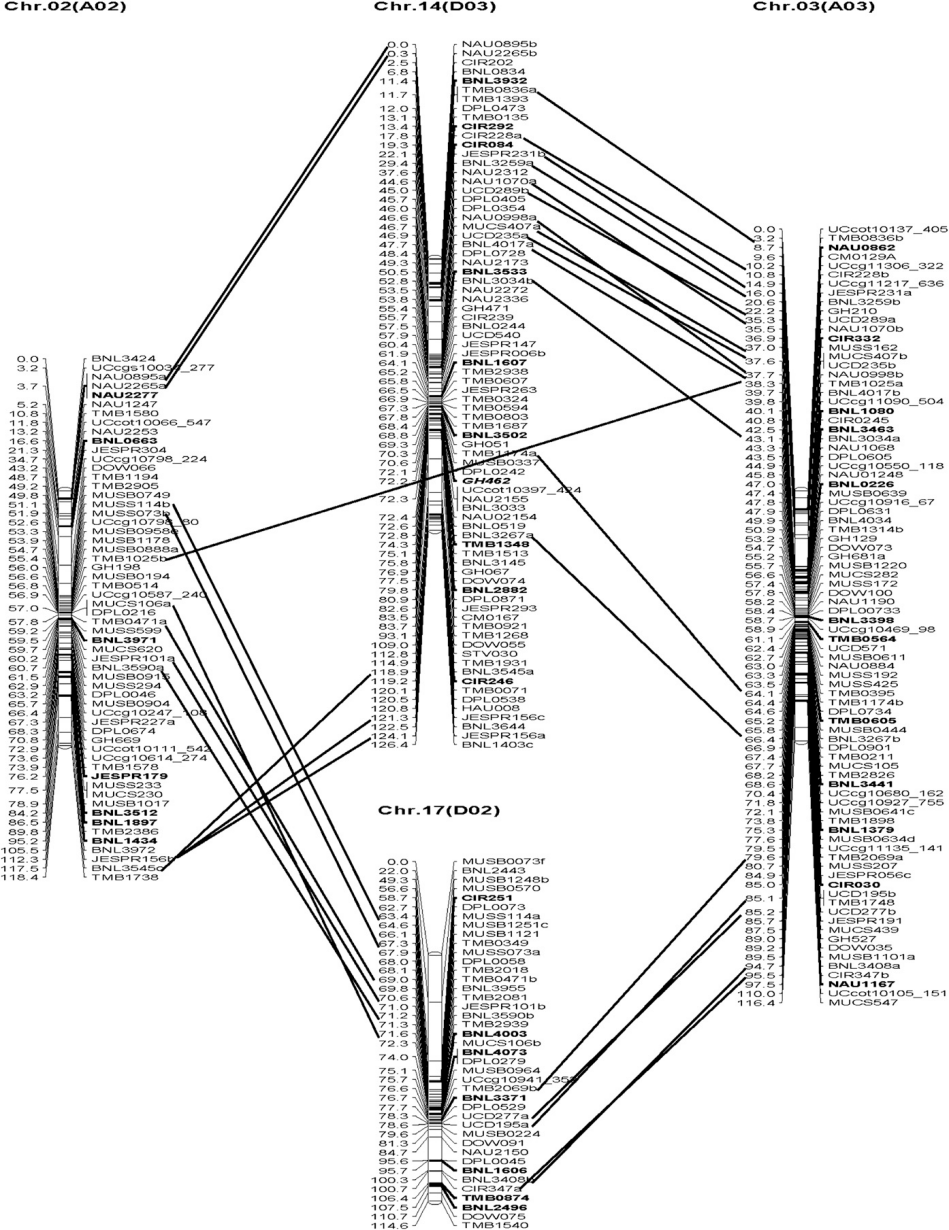

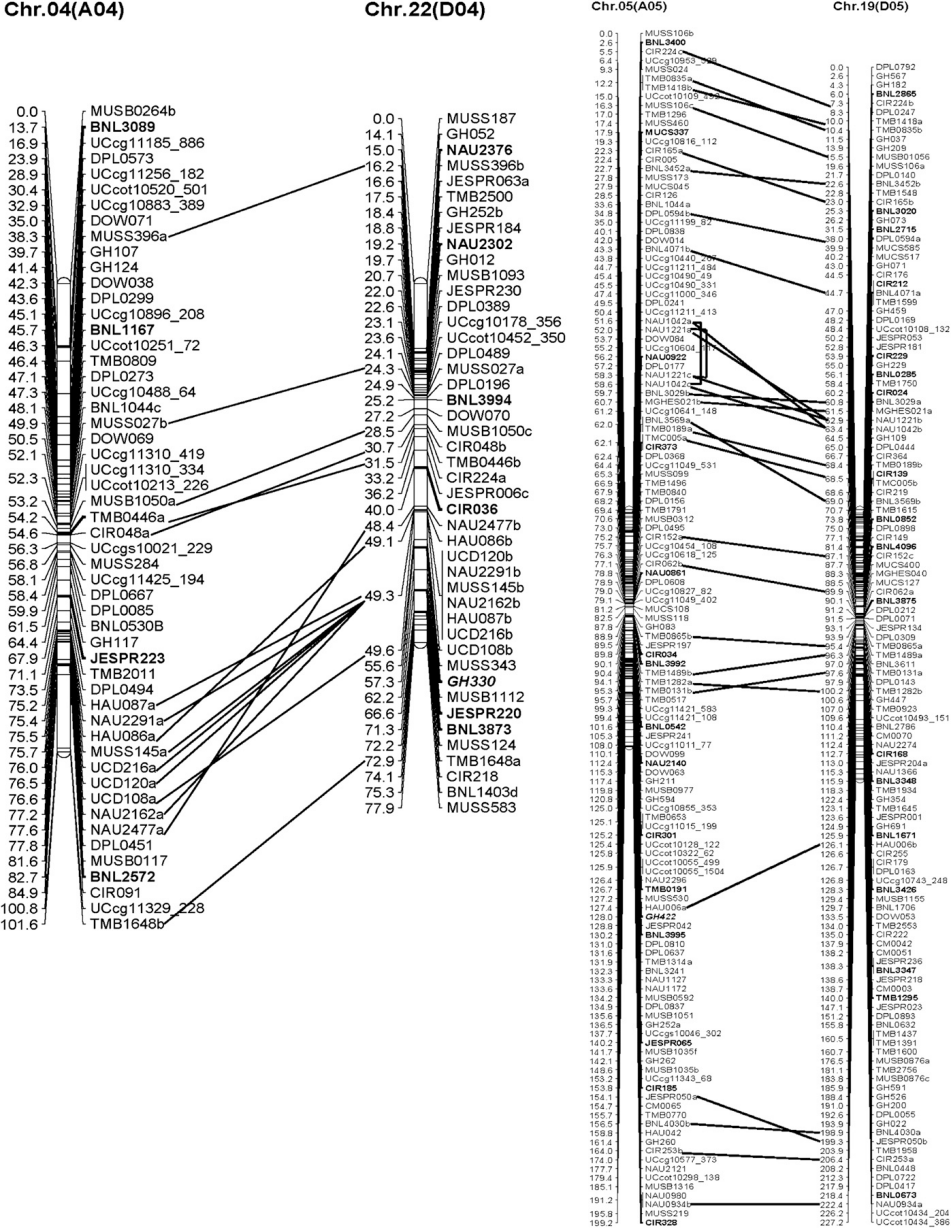

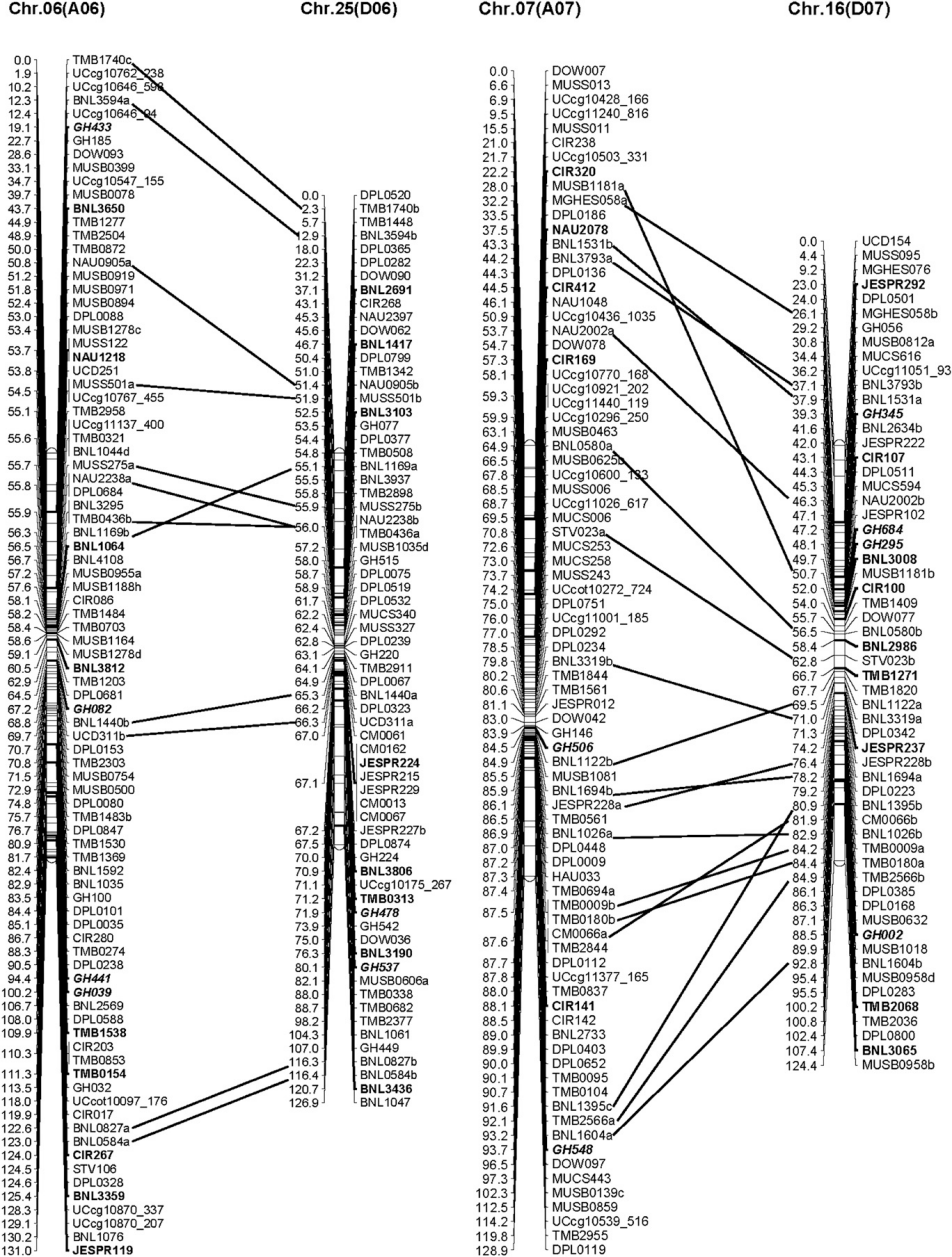

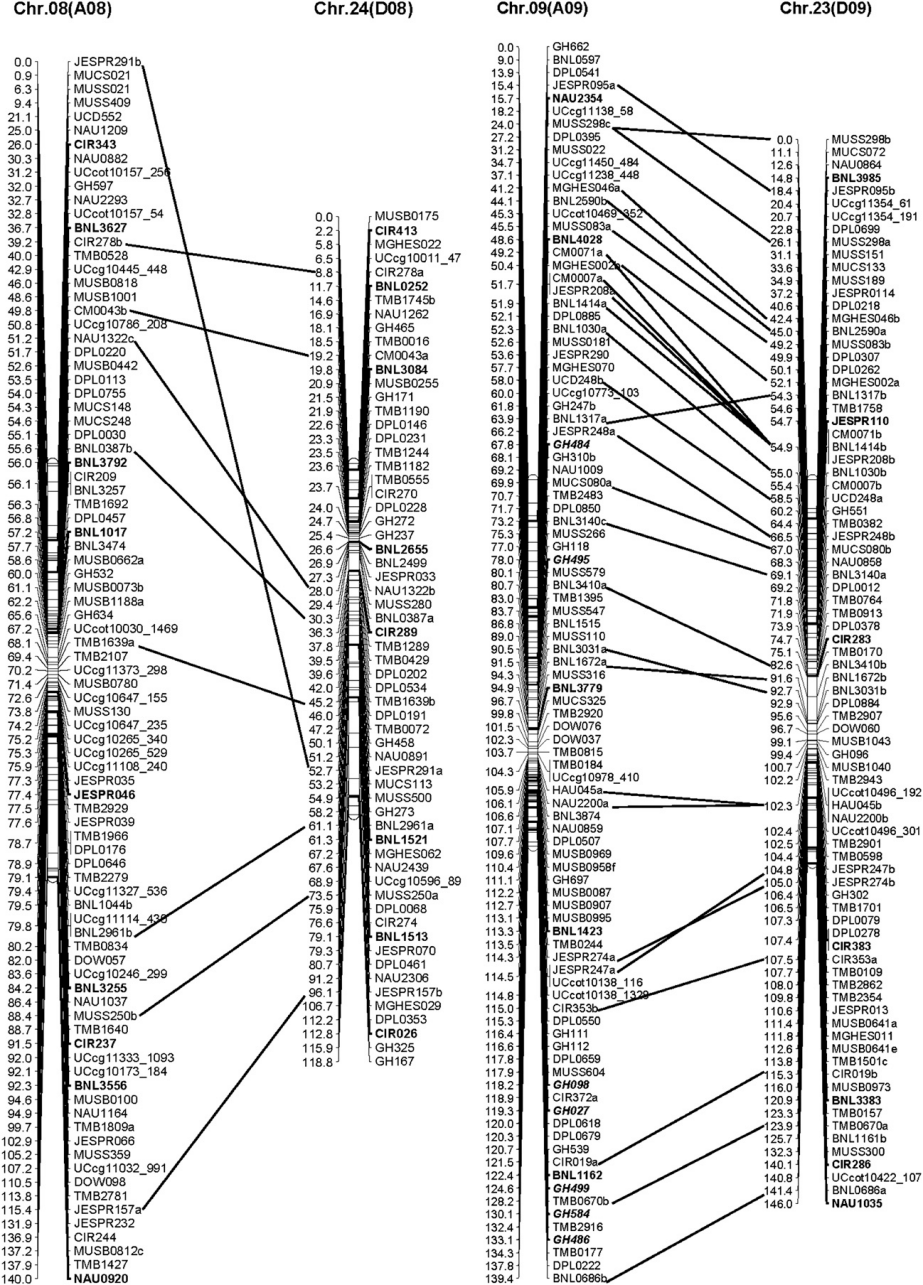

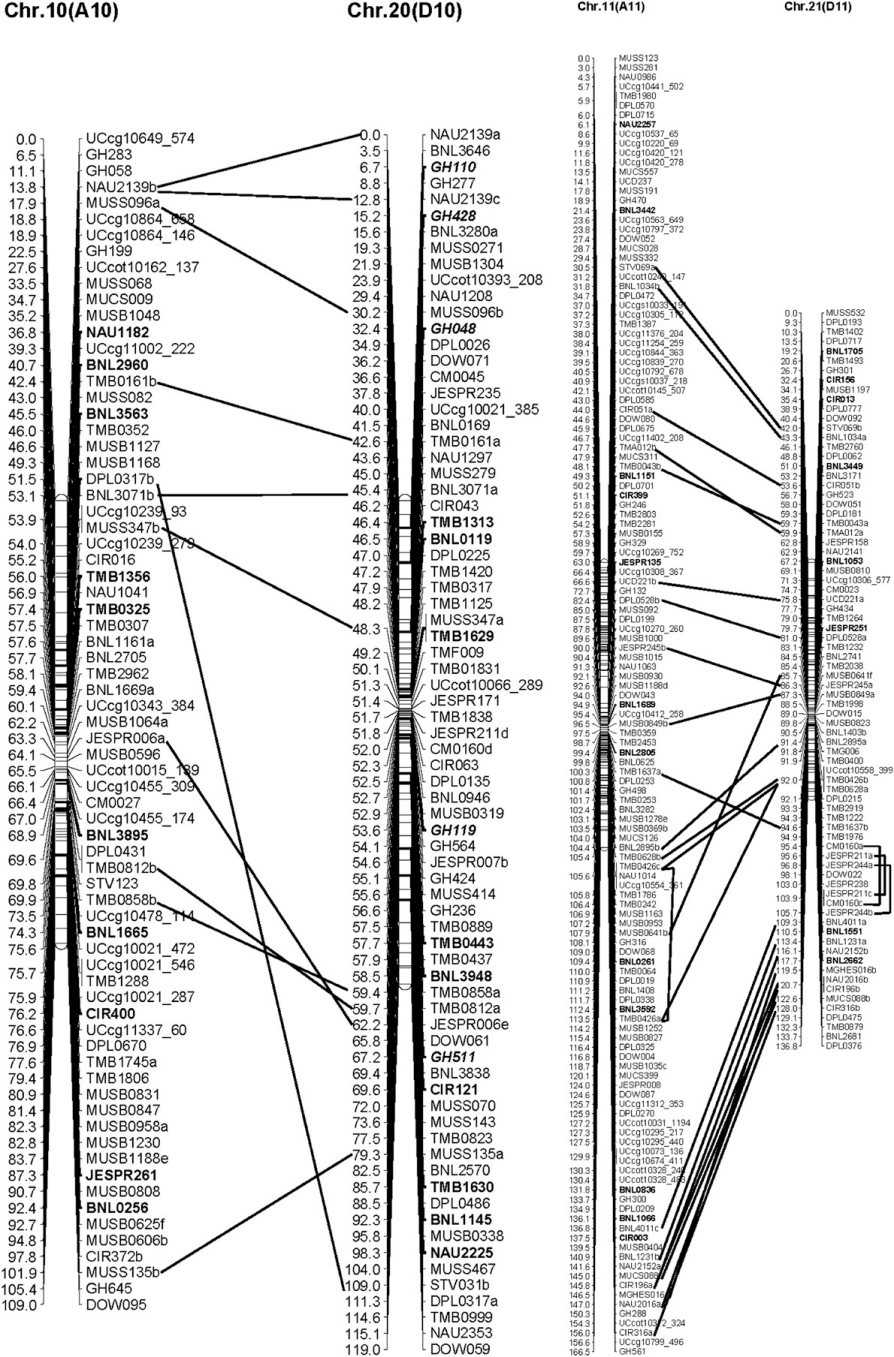

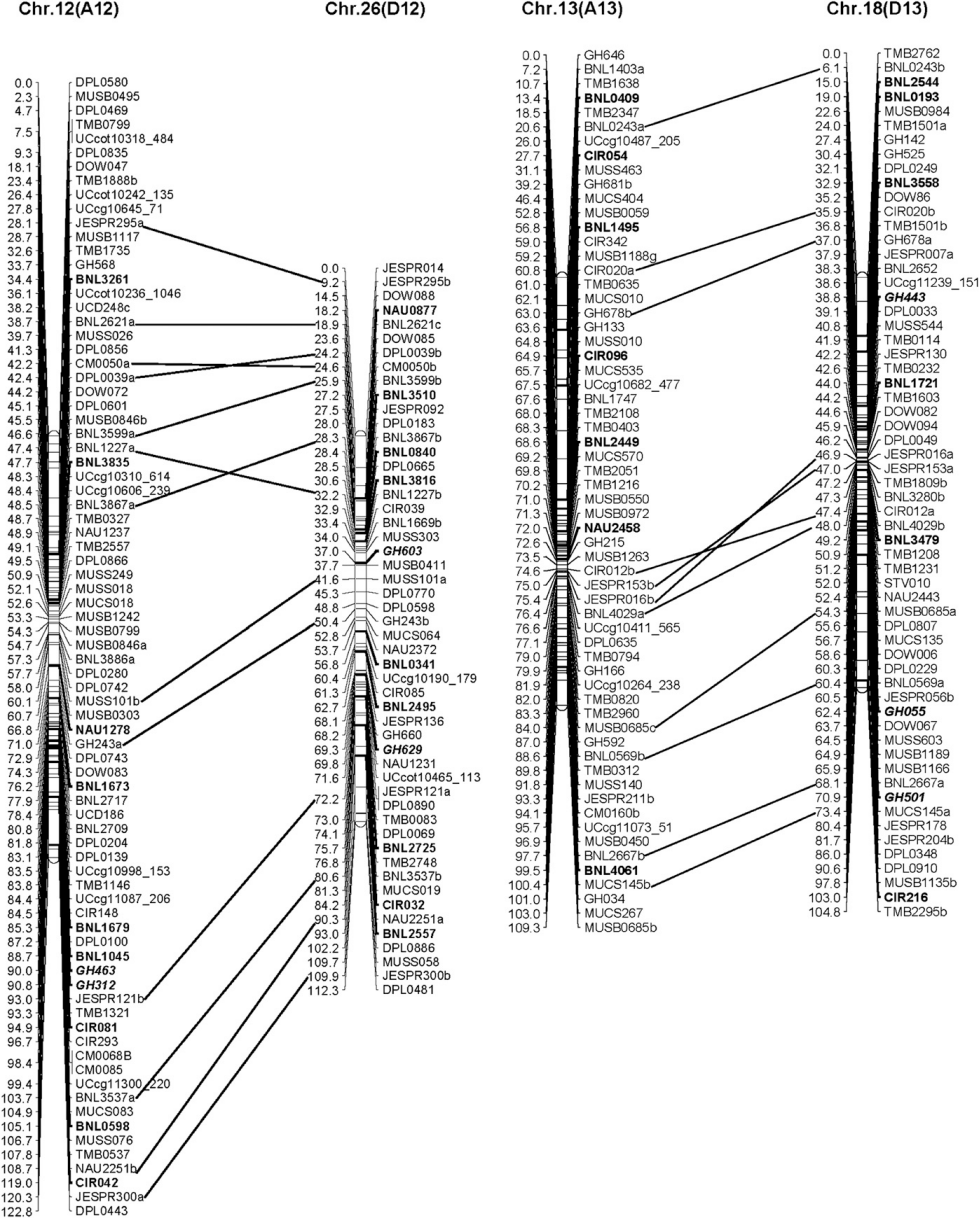
Genetic linkage maps of 26 allotetraploid cotton chromosomes that are presented in 13 At and Dt subgenome homeologous pairs (in parentheses). The names of DNA markers are shown on the right, and the positions of the markers are shown in Kosambi centiMorgan (cM) on the left. A line bar connects duplicate marker loci between a pair of homeologous chromosomes. Marker loci in bold are assigned to cotton chromosomes by previously published studies (Frelichowski Jr. *et al.* 2006; Guo *et al.* 2007, 2008; Lacape *et al.* 2003; Liu *et al.* 2000; Park *et al.* 2005; Yu *et al.* 2011) and marker loci in italic bold are assigned to cotton chromosomes in this study (Table 3). Homeologous marker linkage relationships indicate of reciprocal rearrangement between ancestral forms of Chr. 02 and 03 and/or 14 and 17 relative to each other; they also indicate that centromeric regions are homeologous between Chr. 02 (A02) and Chr. 17 (D02), as well as between Chr. 03 (A03) and Chr. 14 (D03). Intrachromosomal duplications were noted in Chr. 5, 11, and 21, the latter two in homeologous segments.

The genetic linkage map comprises 2072 SSR and SNP loci mapped to the 26 linkage groups, corresponding to 26 chromosomes of allotetraploid cotton, for a total map distance of 3380 cM (Table 2 and Figure 2). The average marker interval in this map is 1.63 cM. Forty-seven pairs of marker loci were found to be either identical or cosegregated (Table S2), and therefore only one locus from each pair is shown on the map. For example, BNL3545b is identical to or cosegregated with BNL3545a, so only BNL3545a is shown on Chr. 14 (D03).

**Table 2:** Distribution of 2072 SSR and SNP marker loci among the 26 allotetraploid cotton chromosomes

Chromosome	No. Marker Loci	Recombinational Size, cM	Average Marker Interval, cM	No. Gaps >10 cM (Largest)
A-subgenome				
Chr.01(A01)	66	144.4	2.19	2 (14.46)
Chr.02(A02)	60	118.4	1.97	1 (13.46)
Chr.03(A03)	87	116.4	1.34	2 (13.09)
Chr.04(A04)	56	101.6	1.81	2 (15.88)
Chr.05(A05)	139	199.2	1.43	1 (10.03)
Chr.06(A06)	89	131.0	1.47	0 (8.35)
Chr.07(A07)	87	128.9	1.48	1 (10.23)
Chr.08(A08)	92	140.0	1.52	2 (16.52)
Chr.09(A09)	99	139.4	1.41	0 (9.00)
Chr.10(A10)	75	109.0	1.45	0 (6.55)
Chr.11(A11)	140	166.5	1.19	0 (9.98)
Chr.12(A12)	84	122.8	1.46	0 (8.79)
Chr.13(A13)	64	109.3	1.71	0 (7.17)
Subtotal-At	1138	1726.8	1.52	11 (16.52)
D-subgenome				
Chr.15(D01)	93	118.0	1.27	1 (10.05)
Chr.17(D02)	42	114.6	2.73	2 (22.01)
Chr.14(D03)	79	126.4	1.60	1 (15.92)
Chr.22(D04)	45	77.9	1.73	1 (14.09)
Chr.19(D05)	132	227.2	1.72	1 (15.78)
Chr.25(D06)	70	126.9	1.81	0 (9.249)
Chr.16(D07)	58	124.4	2.15	2 (17.02)
Chr.24(D08)	62	118.8	1.92	1 (10.47)
Chr.23(D09)	83	146.0	1.76	1 (11.07)
Chr.20(D10)	76	119.0	1.57	0 (5.03)
Chr.21(D11)	80	136.8	1.71	0 (9.27)
Chr.26(D12)	53	112.3	2.12	0 (9.23)
Chr.18(D13)	61	104.8	1.72	0 (7.23)
Subtotal-Dt	934	1653.1	1.77	10 (22.01)
Total	2072	3380	1.63	21 (22.01)

SNP, single nucleotide polymorphism; SSR, simple sequence repeat.

The At subgenome consisted of 1138 marker loci (927 SSR and 211 SNP), and the total genetic distance was 1726.8 cM with an average marker interval of 1.52 cM. The largest chromosome in terms of recombination frequency was Chr. 05 (A05), which spans 199.2 cM with 139 marker loci. The second largest was Chr. 11 (A11), which spans 166.5 cM with 140 loci. The shortest was Chr. 04 (A04), which spans 101.6 cM with 56 loci (Table 2 and Figure 2). In the At subgenome, there were 11 gaps greater than 10 cM, and the largest gap between two loci was 16.52 cM on Chr. 08 (A08).

The Dt subgenome consisted of 934 marker loci (898 SSR and 36 SNP), and the total genetic distance was 1653.1 cM, with an average marker interval of 1.77 cM. The largest chromosome with respect to recombination frequency was Chr. 19 (D05), which spans 227.2 cM with 132 loci, and the shortest chromosome was Chr. 22 (D04), which spans 77.9 cM with 45 loci (Table 2 and Figure 2). There were 10 gaps greater than 10 cM, and the largest gap between two loci was 22.01 cM on Chr. 17 (D02). Although SNP marker loci were largely mapped in the At subgenome because of the A-genome origin of SNP primers (Van Deynze *et al.* 2009), the At subgenome and Dt subgenome had virtually similar numbers of SSR marker loci and total genetic distances. Furthermore, there were similar amounts of recombination between each of 13 pairs of cotton homeologous chromosomes.

### Complete assignment of linkage groups to cotton chromosomes

**Figure 3:**
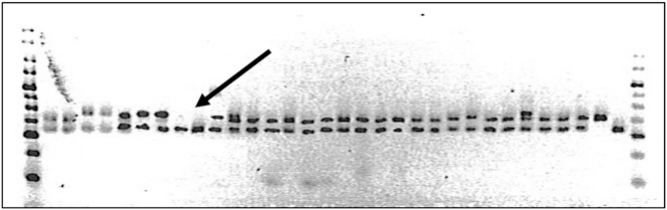
Deletion analysis of cotton SSR markers. GH584 amplified (from L to R) cotton hemizygous F_1_ hypoaneuploids as well as homozygous TM-1 and 3-79. TM-1 allele (140 bp) was missing in both lanes (see arrow) with the H09 template, suggesting the location of GH584 locus on chromosome 09 (A09).

A complete set of 26 cotton chromosomes (13 At subgenome and 13 Dt subgenome) were identified that correspond to 26 respective linkage groups (Figure 2). Assignment of SSR markers and linkage groups to the cotton chromosomes was achieved in part by comparison of the common markers (bold font in Figure 2) with the previous SSR mapping reports (Frelichowski Jr. *et al.* 2006; Guo *et al.* 2007; Lacape *et al.* 2003; Park *et al.* 2005; Yu *et al.* 2011) and with the three aneuploid studies for TMB markers (Guo *et al.* 2008) and BNL markers (Gutiérrez *et al.* 2009; Liu *et al.* 2000), respectively. In addition, hypoaneuploid cottons were also analyzed to identify TM-1 deficiency with 37 newly developed GH markers (bold italic in Figure 2) from *G. hirsutum* and other SSR markers of interest in the mapping study (Table 3 and Figure 3) (Hoffman *et al.* 2007). Although most SSR markers generally agreed with published reports, a few incongruities, such as GH034 and GH526, between various data types were encountered when cotton hypoaneuploid stocks were used along with individual mapping populations. Additional mapping analyses in the present research confirmed or reassigned such SSR markers to the corresponding cotton chromosomes (Table 3).

**Table 3:** Assignment of 37 GH SSR markers to specific allotetraploid cotton chromosomes

Marker Name	Fragment Size, bp		Hypoaneuploid	Mapped Chromosome
	TM-1 allele	3-79 allele		
GH002	75	65	H16	Chr.16(D07)
GH027	70	80	H09	Chr.09(A09)
GH034	130	120	H07	Chr.13(A13)
GH039	125	120	H06	Chr.06(A06)
GH048	90	98	H20	Chr.20(D10)
GH055	175	170	Te18sh	Chr.18(D13)
GH082	175	155	H06	Chr.06(A06)
GH098	130	145	H09	Chr.09(A09)
GH110	105	80	H20	Chr.20(D10)
GH119	150	165	H20	Chr.20(D10)
GH295	95	75	H16	Chr.16(D07)
GH312	110	102	H12	Chr.12(A12)
GH330	105	115	Te22Lo	Chr.22(D04)
GH336	98	86	H01	Chr01(A01)
GH345	115	103	H16	Chr.16(D07)
GH422	116	126	Te5Lo	Chr.05(A05)
GH428	195	170	H20	Chr.20(D10)
GH433	168	150	H06	Chr.06(A06)
GH441	175	150	H06	Chr.06(A06)
GH443	150	120	H18	Chr.18(D13)
GH462	170	152	Te14Lo	Chr.14(D03)
GH463	150	165	H12	Chr.12(A12)
GH478	90	100	H25	Chr.25(D06)
GH484	140	145	H09	Chr.09(A09)
GH486	155	130	H09	Chr.09(A09)
GH495	80	72	H09	Chr.09(A09)
GH499	148	144	H09	Chr.09(A09)
GH501	200	202	H18	Chr.18(D13)
GH506	134	160	H07	Chr.07(A07)
GH511	135	130	H20	Chr.20(D10)
GH526	100	200	Te22Lo	Chr19(D05)
GH537	175	170	H25	Chr.25(D06)
GH548	120	140	H07	Chr.07(A07)
GH584	140	120	H09	Chr.09(A09)
GH603	154	158	H26	Chr.26(D12)
GH629	128	132	H26	Chr.26(D12)
GH684	102	90	H16	Chr.16(D07)

SSR, simple sequence repeat.

### Genomic duplication and chromosomal translocation of allotetraploid cottons

Among 1601 SSR primer pairs that amplified 1895 loci in TM-1 and 3-79, 257 SSR primer pairs amplified two or more loci, resulting in a total of 551 duplicate loci. Excluding dominant loci amplified by these SSRs, there were 494 codominant loci that were duplicated, resulting in 247 pairs (Table S3). Most of the duplicate loci were mapped on the homeologous chromosome pairs (Table 4 and Figure 2). The relative orders of most duplicate loci on the homeologous chromosomes were similar (Figure 2). The duplicate loci identified by these SSR markers demonstrated the complex but linear features of the allotetraploid cotton genomes. A few duplicate loci also were present between nonhomeologous chromosomes and/or within the same subgenome, which indicated likely genome rearrangements (Table 4 and Table S3). For example, an intrasubgenome duplication was revealed by the marker BNL1044 between Chr. 04 (A04) and Chr. 05 (A05). Distinct intrachromosome duplications were indicated by one SSR duplication in Chr. 11 (A11) and three SSRs in Chr. 21 (D11) (Figure 2). In chromosome 11 (A11), TMB0426 revealed two loci that were mapped 8.1 cM apart. In chromosome 21 (D11), three markers (*i.e.* CM0160, JESPR211, and JESPR244) each revealed two loci. In the latter, the recombination rates remained similar (~8-9 cM) but the relative orders among duplicated loci were altered.

**Table 4:** Pairs of duplicate marker loci between homeologous and nonhomeologous chromosomes in cotton

Homeologous Chromosomes	No. Pairs of Duplicate Loci	Nonhomeologous Chromosomes	No. Pairs of Duplicate Loci
Chr.01(A01)-Chr.15(D01)	19	Chr.02(A02)-Chr.14(D03)	5
Chr.02(A02)-Chr.17(D02)	6	Chr.03(A03)-Chr.17(D02)	5
Chr.03(A03)-Chr.14(D03)	13	Chr.02(A02)-Chr.03(A03)	1
Chr.04(A04)-Chr.22(D04)	15	Chr.04(A04)-Chr.05(A05)	1
Chr.05(A05)-Chr.19(D05)	27	Chr.05(A05)-Chr.22(D04)	1
Chr.06(A06)-Chr.25(D06)	12		
Chr.07(A07)-Chr.16(D07)	18		
Chr.08(A08)-Chr.24(D08)	9		
Chr.09(A09)-Chr.23(D09)	27		
Chr.10(A10)-Chr.20(D10)	10		
Chr.11(A11)-Chr.21(D11)	24		
Chr.12(A12)-Chr.26(D12)	13		
Chr.13(A13)-Chr.18(D13)	11		
Totals	204		13

A postpolyploidization reciprocal translocation of chromosomes 02 (A02) and 03 (A03) was suggested by 10 pairs of duplicate loci (Figure 2 and Table 4). Five pairs of duplicate loci were identified between chromosomes 02 (A02) and 14 (D03) and 5 pairs between chromosomes 03 (A03) and 17 (D02). The marker TMB1025 revealed duplicate loci between chromosomes 02 (A02) and 03 (A03), which inferred a possible breakpoint for the reciprocal translocation in these two At subgenome chromosomes. Additional mapping data in the vicinity of TMB1025 will be necessary to confirm this conclusion. Another translocation between At subgenome chromosomes 04 (A04) and 05 (A05), as previously suggested by Guo *et al.* (2007), was observed by the marker BNL1044 loci (BNL1044a at 33.6 cM of A05) and (BNL1044c at 48.1 cM of A04) (Figure 2 and Table S3). Furthermore, the marker GH252 loci showed a translocation between non-homeologous chromosomes 05 (A05) with GH252a at 136.4 cM and 22 (D04) with GH252b at 18.3 cM.

## Discussion

The high-density genetic linkage map created in this research is composed of 2072 SSR and SNP loci representing many individual groups of the cotton research community, and it provides a transferable platform that is essential for a broad spectrum of basic and applied studies aimed at understanding and manipulating complex cotton genomes. Among the 17 sets of SSR and SNP marker loci, BAC-derived SSRs (310 TMB Table S1 and 155 MUSB) facilitate an integration of genetic and physical maps of the cotton chromosomes (Frelichowski Jr. *et al.* 2006; Xu *et al.* 2008). The markers linked to the novel genes can be used to screen cotton BAC clones or physical contigs from which the SSR markers were developed (Yin *et al.* 2006). The 357 EST-derived SSR markers mapped herein offer an opportunity to study functional genes and gene islands for fiber development and other important traits of interest. In addition, the genetic mapping of the 247 SNP markers is the first major public effort to use nucleotide sequence diversity in cotton species by mapping SNP loci (Table S1). Localization of these SNP markers to the 26 individual cotton chromosomes and their integration with large numbers of SSR markers will facilitate other studies in cotton genomics. We believe that the high-density genetic map reported herein is a saturated one for the allotetraploid cotton, as evidenced by a separate mapping analysis (data not shown). Further increase in the map density may not significantly change the total genetic length of this map but will facilitate whole-genome physical alignment, sequencing, and mapping of genes for cotton improvement.

Deviation from a Mendelian segregation ratio is common in intra- and interspecific crosses (Causse *et al.* 1994; Lacape *et al.* 2009; Rong *et al.* 2004; Ulloa *et al.* 2002; Yu *et al.* 2011). An extremely severe distortion (99%) toward *G. hirsutum* was observed by Lacape *et al.* (2009) when 140 RILs were used to produce a low-density map of approximately 800 loci. Only 15 of the 140 RILs exhibited 50% or more *G. barbadense* parental alleles. In this research, TM-1 was less environmentally sensitive than 3-79, as reflected by the allele transmission preference in the advancement of generations of the RIL population (Figure 1). Of the 2072 mapped marker loci, 1391 (67.1%) fit an expected 1:1 segregation ratio, and 681 (32.9%) deviated significantly (χ^2^ > 3.8) from expectations among 186 RILs. The 681 segregation-distorted loci (SDL) were mapped in all 26 groups with 349 mapped in At subgenome, and 332 in Dt subgenome chromosomes. Four chromosomes, *i.e.* Chr. 15 (D01), Chr. 05 (A05), Chr. 07 (A07), and Chr. 08 (A08), had the most SDL, with 68, 60, 57, and 42 loci, respectively. However, Chr. 26 (D12) has the greatest percentage of SDL, 75%, followed by Chr. 15 (D01) with 73.9%, Chr. 07 (A07) with 68.7% and Chr. 05 (A05) with 45.1%. In most cases, the SDL were mapped at centromeric regions.

Our mapping studies indicate that the two subgenomes of allotetraploid cottons are equivalent in recombination frequencies despite the extra repetitive DNA in the At subgenome (Zhao *et al.* 1998). This result is consistent with other independent mapping studies in which the authors used different allotetraploid cotton populations (F_2_ or BC_1_) where variation between At and Dt map sizes supports the ratio of our genetic distances between the two subgenomes. Rong *et al.* (2004) mapped a total of 2584 STS loci that span 4447 cM, with the A subgenome being 9.5% larger genetically than the D subgenome. To the contrary, Guo *et al.* (2007) mapped a total of 1790 SSR loci that span 3426 cM, with the D subgenome being 4.5% larger genetically than the A subgenome. Yu *et al.* (2011) mapped a total of 2316 SSR loci that span 4419 cM with the A subgenome being 3.9% larger genetically than the D subgenome. In this study, the tetraploid cotton were mapped with 1106 loci (54.5%) on 13 At chromosomes at 1726.8 cM (51.1%) and 922 loci (45.5%) on 13 Dt chromosomes at 1653.1 cM (48.9%). Variation in the ratio of subgenome map distances is likely the result of differences in mapping population sizes, as well as in the numbers and sources of DNA markers.

As evidenced in our mapping data, two reciprocal translocations (between Chr. 02 and 03 and between Chr. 04 and 05) are inferred during or after the polyploidization process of two ancestral diploid genomes (A and D). The translocation breakpoint between Chr. 02 and Chr. 03 may be at or near homeologous SSR marker TMB1025. Further investigation is needed to identify additional markers in the vicinity of TMB1025. On the basis of homeologous markers of the two chromosome pairs (A02-D02 and A03-D03), the majority of duplicate loci were mapped to individual pairs of Chr. 02 *vs.* Chr. 17 and Chr. 03 *vs.* Chr. 14. The centromeric cores of these chromosomes seem to show the homeologous relationship, either reciprocal insertional translocations or two temporally separate traditional reciprocal translocations. Thus, we propose to name Chr. 17 as D02 and Chr. 14 as D03, whereas Chr. 02 and Chr. 03 remain as A02 and A03, respectively, which is a revision to Wang *et al.* (2006b) and Guo *et al.* (2007). Duplication of marker loci revealed genome rearrangements within the same individual chromosomes and/or between nonhomeologous chromosomes (Table S3). We recognize that nomenclature revision of cotton chromosomes and linkage groups would be needed in the future, but this could be accomplished by an international committee of experts in the subject matter.

Genetic mapping coupled with physical alignment of genomic regions into chromosomal maps will expedite the discovery of resistance (R) or pathogen-induced R genes underlying QTL involved in resistance to nematode and *Fusarium* wilt (Ulloa *et al.* 2011). Chromosomes 11 (A11) and 21 (D11) are homologs that harbor important genes for cotton improvement because these chromosomes contain genes for resistance to reniform (Dighe *et al.* 2009) and root-knot nematodes (Wang *et al.* 2006a), race 1 of *Fusarium* (Ulloa *et al.* 2010) and other traits affecting fiber yield and quality. The high-density genetic map will facilitate and expedite the analysis of plant defense genes against nematodes and other biotrophic pathogens.

This high-density cotton map was constructed with an immortal RIL mapping population. A high level of homozygosity in this RIL population (currently in F_8_-F_9_) was achieved with less than 5% genome-wide residual heterozygosity. The RILs are maintained as living stocks to produce seed sources for multilocation research on fiber among other traits and to extract fresh DNA samples for a broad spectrum of genomic studies. Our mapping population of 186 RILs is the largest population ever used in high-density cotton genetic mapping. The accuracy of mapping results can be improved substantially as the proportion of recombination between the two linked markers in an inbred population is about twice that of a single meiotic event F_2_ or BC_1_ population when linkage distances are small (<12.5 cM) and increase nonlinearly to 50% for unlinked markers (Burr *et al.* 1988; Haldane and Waddington 1931). This population provides the greatest mapping power currently known in cotton to detect additional loci between closely linked markers by members of the cotton research community who are interested in SSR and SNP augmentation. The advantages of this immortal RIL population and its parental lines make it practical for high-resolution consensus mapping with additional sequence-based portable markers, enabling better understanding and exploitation of complex *Gossypium* genomes (Mace *et al.* 2009). This information will complement other work because of the use of the same parents in developing genetic resources, such as hypoaneuploid cytogenetic stocks, chromosome substitution lines, chromosome specific RILs, and QTL mapping populations in other research programs (Jenkins *et al.* 2006, 2007; Saha *et al.* 2006, 2010, 2011; Stelly *et al.* 2005).

The International Cotton Genome Initiative (http://icgi.tamu.edu/) has proposed to map and sequence the *Gossypium* genomes (Brubaker *et al.* 2000; Chen *et al.* 2007; Paterson 2008; Wilkins 2008; Yu *et al.* 2008), but large amounts of dispersed repetitive elements and duplicate loci between and within the allotetraploid cotton chromosomes present great challenges to properly assemble a complex *Gossypium* genome. Development of additional numbers of SSR and SNP markers from the fingerprinted and sequenced BAC clones or physical contigs, such as the 310 TMB and 155 MUSB markers on the present map, would provide a unique opportunity to facilitate the mapping the gaps (5−15 cM) of genomic regions (Lin *et al.* 2010; Xu *et al.* 2008; Yin *et al.* 2006). A high-density genetic map is essential in the reconciliation with a whole-genome physical map to facilitate genome sequencing, sequence assembly, gene mapping, and the design of targeted genetic markers for better understanding and improvement of the cotton plant.

## Supplementary Material

Supporting Information
