# Supplementary material for: A High-Density Simple Sequence Repeat and Single Nucleotide Polymorphism Genetic Map of the Tetraploid Cotton Genome
Source: G3 (Bethesda). 2012 Jan 1;2(1):43–58. doi: 10.1534/g3.111.001552 (PMC3276184; doi:10.1534/g3.111.001552)
Supplement: Supporting Information [file supp_2_1_43__index.html]

Supporting Information 

# A High-Density Simple Sequence Repeat and Single Nucleotide Polymorphism Genetic Map of the Tetraploid Cotton Genome

## Supporting Information for Yu *et al.*, 2012

**Files in this Data Supplement:**

- Supporting Information - Tables S1-S3 (PDF, 140 KB)
- Table S1 - Distribution of 247 SNP and 310 TMB markers among the 26 chromosomes (PDF, 52 KB)
- Table S2 - SSR and SNP marker loci that are either Identical or co-segregated in the TM-1 x 3-79 RIL mapping population (PDF, 52 KB)
- Table S3 - Two hundred forty seven pairs of duplicate SSR loci and their chromosome locations (PDF, 128 KB)
