# Supplementary material for: A High-Density Simple Sequence Repeat and Single Nucleotide Polymorphism Genetic Map of the Tetraploid Cotton Genome
Source: G3 (Bethesda). 2012 Jan 1;2(1):43–58. doi: 10.1534/g3.111.001552 (PMC3276184; doi:10.1534/g3.111.001552)
Supplement: Supporting Information [file supp_2.1.43_001552SI.pdf]

Table S1 Distribution of 247 SNP and 310 TMB markers among the 26 chromosomes.

Chromosome	# Markers		Chromosome	# Markers	
	SNP	TMB		SNP	TMB
Chr.01(A01)	4	12	Chr.15(D01)	4	17
Chr.02(A02)	10	9	Chr.17(D02)	1	8
Chr.03(A03)	12	12	Chr.14(D03)	2	16
Chr.04(A04)	16	4	Chr.22(D04)	2	3
Chr.05(A05)	35	17	Chr.19(D05)	7	22
Chr.06(A06)	10	18	Chr.25(D06)	1	12
Chr.07(A07)	19	12	Chr.16(D07)	1	8
Chr.08(A08)	20	12	Chr.24(D08)	2	10
Chr.09(A09)	10	9	Chr.23(D09)	5	16
Chr.10(A10)	19	11	Chr.20(D10)	4	17
Chr.11(A11)	36	16	Chr.21(D11)	3	18
Chr.12(A12)	13	8	Chr.26(D12)	3	2
Chr.13(A13)	7	11	Chr.18(D13)	1	10
Subtotal-At	211	151	Subtotal-Dt	36	159

Table S2 SSR and SNP marker loci that are either Identical or co-segregated in the TM-1 x 3-79 RIL mapping population.

#	Marker locus 1 identical to	Marker locus 2
1	BNL3545a	BNL3545b
2	DPL0031a	UCcg10265_529
3	DPL0215	UCcot10558_399
4	DPL0264	DPL0854
5	DPL0301	TMB0436a
6	JESPR215	JESPR229
7	NAU0934b	NAU0980
8	UCcg10010_234	UCcg10010_475
9	UCcg10295_217	UCcot10031_405
10	UCcg10310_614	UCcg10809_201
11	UCcg10436_1035	UCcg10436_1135
12	UCcg10436_1035	UCcg10436_861
13	UCcg10503_331	UCcg10503_86
14	UCcg10614_274	UCcg10614_520
15	UCcg10682_477	UCcg10682_712
16	UCcg10770_168	UCcg10770_48
17	UCcg10773_103	UCcg10773_312
18	UCcg10797_372	UCcg10797_669
19	UCcg10816_112	UCcg10816_726
20	UCcg10855_353	UCcg10855_642
21	UCcg10883_389	UCcg10883_495
22	UCcg10998_153	UCcg10998_265
23	UCcg11002_222	UCcg11002_302
24	UCcg11002_222	UCcot10019_389
25	UCcg11015_199	UCcg11015_757
26	UCcg11073_51	UCcg11073_768
27	UCcg11135_141	UCcg11135_438
28	UCcg11256_182	UCcg11256_354
29	UCcg11440_119	UCcg11440_818
30	UCcgs10034_277	UCcgs10034_82
31	UCcot10030_1469	UCcot10030_820
32	UCcot10031_1194	UCcot10112_120
33	UCcot10031_1194	UCcot10112_228

34	UCcot10055_499	UCcot10055_591
35	UCcot10112_120	UCcot10112_228
36	UCcot10128_122	UCcot10128_269
37	UCcot10138_116	UCcot10138_775
38	UCcot10157_256	UCcot10157_97
39	UCcot10162_137	UCcot10162_64
40	UCcot10213_226	UCcot10213_61
41	UCcot10242_135	UCcot10242_76
42	UCcot10318_484	UCcot10318_588
43	UCcot10393_208	UCcot10393_558
44	UCcot10397_424	UCcot10397_643
45	UCcot10434_204	UCcot10434_536
46	UCcot10465_113	UCcot10465_333
47	UCcot10493_151	UCcot10493_618

Table S3 Two hundred forty seven pairs of duplicate SSR loci and their chromosome locations.

#	Marker locus	Homeologous relationship	Chromosome	Position (cM)
1	BNL1350a	Chr.01—Chr.15	Chr.01(A01)	57.334
2	BNL1350b	Chr.01—Chr.15	Chr.15(D01)	58.606
3	BNL1667a	Chr.01—Chr.15	Chr.01(A01)	34.763
4	BNL1667b	Chr.01—Chr.15	Chr.15(D01)	41.505
5	BNL3090a	Chr.01—Chr.15	Chr.15(D01)	51.859
6	BNL3090b	Chr.01—Chr.15	Chr.01(A01)	47.641
7	CIR009a	Chr.01—Chr.15	Chr.15(D01)	114.214
8	CIR009b	Chr.01—Chr.15	Chr.01(A01)	125.895
9	DPL0887a	Chr.01—Chr.15	Chr.01(A01)	53.813
10	DPL0887b	Chr.01—Chr.15	Chr.15(D01)	54.685
11	GH649a	Chr.01—Chr.15	Chr.01(A01)	57.709
12	GH649b	Chr.01—Chr.15	Chr.15(D01)	57.355
13	JESPR063b	Chr.01—Chr.15	Chr.01(A01)	59.809
14	JESPR063c	Chr.01—Chr.15	Chr.15(D01)	65.002
15	JESPR240a	Chr.01—Chr.15	Chr.01(A01)	62.674
16	JESPR240b	Chr.01—Chr.15	Chr.15(D01)	64.413
17	MGHES010a	Chr.01—Chr.15	Chr.01(A01)	62.855
18	MGHES010b	Chr.01—Chr.15	Chr.15(D01)	64.48
19	MUCS084a	Chr.01—Chr.15	Chr.15(D01)	64.223
20	MUCS084b	Chr.01—Chr.15	Chr.01(A01)	61.908
21	MUCS164a	Chr.01—Chr.15	Chr.15(D01)	115.532
22	MUCS164c	Chr.01—Chr.15	Chr.01(A01)	107.524
23	MUSS161a	Chr.01—Chr.15	Chr.15(D01)	117.409
24	MUSS161c	Chr.01—Chr.15	Chr.01(A01)	129.911
25	MUSS422a	Chr.01—Chr.15	Chr.15(D01)	64.255
26	MUSS422b	Chr.01—Chr.15	Chr.01(A01)	62.27
27	NAU2437a	Chr.01—Chr.15	Chr.01(A01)	144.377
28	NAU2437b	Chr.01—Chr.15	Chr.15(D01)	117.997
29	TMB0119a	Chr.01—Chr.15	Chr.01(A01)	34.095
30	TMB0119b	Chr.01—Chr.15	Chr.15(D01)	42.663
31	TMB0283a	Chr.01—Chr.15	Chr.15(D01)	44.688
32	TMB0283b	Chr.01—Chr.15	Chr.01(A01)	38.977
33	TMB0301a	Chr.01—Chr.15	Chr.01(A01)	71.892
34	TMB0301b	Chr.01—Chr.15	Chr.15(D01)	67.873
35	TMB1181a	Chr.01—Chr.15	Chr.15(D01)	45.024
36	TMB1181b	Chr.01—Chr.15	Chr.01(A01)	39.397
37	TMB1869a	Chr.01—Chr.15	Chr.15(D01)	60.304
38	TMB1869b	Chr.01—Chr.15	Chr.01(A01)	63.106
39	BNL3590a	Chr.02--Chr.17	Chr.02(A02)	60.681
40	BNL3590b	Chr.02--Chr.17	Chr.17(D02)	43.389
41	JESPR101a	Chr.02--Chr.17	Chr.02(A02)	60.154
42	JESPR101b	Chr.02--Chr.17	Chr.17(D02)	43.568
43	MUCS106a	Chr.02--Chr.17	Chr.02(A02)	56.994
44	MUCS106b	Chr.02--Chr.17	Chr.17(D02)	42.323
45	MUSS073a	Chr.02--Chr.17	Chr.17(D02)	46.737

46	MUSS073b	Chr.02--Chr.17	Chr.02(A02)	51.916
47	MUSS114a	Chr.02--Chr.17	Chr.17(D02)	51.223
48	MUSS114b	Chr.02--Chr.17	Chr.02(A02)	51.077
49	TMB0471a	Chr.02--Chr.17	Chr.02(A02)	57.791
50	TMB0471b	Chr.02--Chr.17	Chr.17(D02)	45.637
51	BNL3034a	Chr.03--Chr.14	Chr.03(A03)	43.075
52	BNL3034b	Chr.03--Chr.14	Chr.14(D03)	52.798
53	BNL3259a	Chr.03--Chr.14	Chr.14(D03)	29.372
54	BNL3259b	Chr.03--Chr.14	Chr.03(A03)	20.588
55	BNL3267a	Chr.03--Chr.14	Chr.14(D03)	72.832
56	BNL3267b	Chr.03--Chr.14	Chr.03(A03)	66.447
57	BNL4017a	Chr.03--Chr.14	Chr.14(D03)	47.691
58	BNL4017b	Chr.03--Chr.14	Chr.03(A03)	39.661
59	CIR228a	Chr.03--Chr.14	Chr.14(D03)	17.808
60	CIR228b	Chr.03--Chr.14	Chr.03(A03)	10.815
61	JESPR231a	Chr.03--Chr.14	Chr.03(A03)	15.992
62	JESPR231b	Chr.03--Chr.14	Chr.14(D03)	22.131
63	MUCS407a	Chr.03--Chr.14	Chr.14(D03)	46.65
64	MUCS407b	Chr.03--Chr.14	Chr.03(A03)	37.565
65	NAU0998a	Chr.03--Chr.14	Chr.14(D03)	46.55
66	NAU0998b	Chr.03--Chr.14	Chr.03(A03)	37.714
67	NAU1070a	Chr.03--Chr.14	Chr.14(D03)	44.591
68	NAU1070b	Chr.03--Chr.14	Chr.03(A03)	35.466
69	TMB0836a	Chr.03--Chr.14	Chr.14(D03)	11.681
70	TMB0836b	Chr.03--Chr.14	Chr.03(A03)	3.234
71	TMB1174a	Chr.03--Chr.14	Chr.14(D03)	70.262
72	TMB1174b	Chr.03--Chr.14	Chr.03(A03)	64.394
73	UCD235a	Chr.03--Chr.14	Chr.14(D03)	46.853
74	UCD235b	Chr.03--Chr.14	Chr.03(A03)	37.615
75	UCD289b	Chr.03--Chr.14	Chr.14(D03)	44.975
76	UCD289a	Chr.03--Chr.14	Chr.03(A03)	35.314
77	CIR048a	Chr.04--Chr.22	Chr.04(A04)	54.629
78	CIR048b	Chr.04--Chr.22	Chr.22(D04)	30.7
79	HAU086a	Chr.04--Chr.22	Chr.04(A04)	75.529
80	HAU086b	Chr.04--Chr.22	Chr.22(D04)	49.101
81	HAU087a	Chr.04--Chr.22	Chr.04(A04)	75.203
82	HAU087b	Chr.04--Chr.22	Chr.22(D04)	49.337
83	MUSB1050a	Chr.04--Chr.22	Chr.04(A04)	53.185
84	MUSB1050c	Chr.04--Chr.22	Chr.22(D04)	28.543
85	MUSS027a	Chr.04--Chr.22	Chr.22(D04)	24.338
86	MUSS027b	Chr.04--Chr.22	Chr.04(A04)	49.924
87	MUSS145a	Chr.04--Chr.22	Chr.04(A04)	75.724
88	MUSS145b	Chr.04--Chr.22	Chr.22(D04)	49.327
89	MUSS396a	Chr.04--Chr.22	Chr.04(A04)	38.294
90	MUSS396b	Chr.04--Chr.22	Chr.22(D04)	16.222
91	NAU2162a	Chr.04--Chr.22	Chr.04(A04)	77.193
92	NAU2162b	Chr.04--Chr.22	Chr.22(D04)	49.332
93	NAU2291a	Chr.04--Chr.22	Chr.04(A04)	75.379
94	NAU2291b	Chr.04--Chr.22	Chr.22(D04)	49.302
95	NAU2477a	Chr.04--Chr.22	Chr.04(A04)	77.602

96	NAU2477b	Chr.04--Chr.22	Chr.22(D04)	48.409
97	TMB0446a	Chr.04--Chr.22	Chr.04(A04)	54.23
98	TMB0446b	Chr.04--Chr.22	Chr.22(D04)	31.504
99	TMB1648a	Chr.04--Chr.22	Chr.22(D04)	72.924
100	TMB1648b	Chr.04--Chr.22	Chr.04(A04)	101.582
101	UCD108a	Chr.04--Chr.22	Chr.04(A04)	76.637
102	UCD108b	Chr.04--Chr.22	Chr.22(D04)	49.573
103	UCD120a	Chr.04--Chr.22	Chr.04(A04)	76.454
104	UCD120b	Chr.04--Chr.22	Chr.22(D04)	49.277
105	UCD216a	Chr.04--Chr.22	Chr.04(A04)	76.02
106	UCD216b	Chr.04--Chr.22	Chr.22(D04)	49.343
107	BNL3029a	Chr.05--Chr.19	Chr.19(D05)	60.77
108	BNL3029b	Chr.05--Chr.19	Chr.05(A05)	59.68
109	BNL3452a	Chr.05--Chr.19	Chr.05(A05)	22.736
110	BNL3452b	Chr.05--Chr.19	Chr.19(D05)	22.592
111	BNL3569a	Chr.05--Chr.19	Chr.05(A05)	62.01
112	BNL3569b	Chr.05--Chr.19	Chr.19(D05)	68.968
113	BNL4030a	Chr.05--Chr.19	Chr.19(D05)	198.939
114	BNL4030b	Chr.05--Chr.19	Chr.05(A05)	156.511
115	BNL4071a	Chr.05--Chr.19	Chr.19(D05)	44.665
116	BNL4071b	Chr.05--Chr.19	Chr.05(A05)	43.337
117	CIR062a	Chr.05--Chr.19	Chr.19(D05)	89.932
118	CIR062b	Chr.05--Chr.19	Chr.05(A05)	77.051
119	CIR152a	Chr.05--Chr.19	Chr.05(A05)	75.178
120	CIR152c	Chr.05--Chr.19	Chr.19(D05)	87.141
121	CIR165a	Chr.05--Chr.19	Chr.05(A05)	22.324
122	CIR165b	Chr.05--Chr.19	Chr.19(D05)	22.993
123	CIR224b	Chr.05--Chr.19	Chr.19(D05)	7.277
124	CIR224c	Chr.05--Chr.19	Chr.05(A05)	5.539
125	CIR253a	Chr.05--Chr.19	Chr.19(D05)	206.415
126	CIR253b	Chr.05--Chr.19	Chr.05(A05)	163.984
127	DPL0594a	Chr.05--Chr.19	Chr.19(D05)	37.99
128	DPL0594b	Chr.05--Chr.19	Chr.05(A05)	34.752
129	HAU006a	Chr.05--Chr.19	Chr.05(A05)	127.409
130	HAU006b	Chr.05--Chr.19	Chr.19(D05)	126.114
131	JESPR050a	Chr.05--Chr.19	Chr.05(A05)	154.073
132	JESPR050b	Chr.05--Chr.19	Chr.19(D05)	199.276
133	MGHES021a	Chr.05--Chr.19	Chr.19(D05)	61.467
134	MGHES021b	Chr.05--Chr.19	Chr.05(A05)	60.684
135	MUSS106a	Chr.05--Chr.19	Chr.19(D05)	19.568
136	MUSS106c	Chr.05--Chr.19	Chr.05(A05)	16.28
137	NAU0934a	Chr.05--Chr.19	Chr.19(D05)	222.381
138	NAU0934b	Chr.05--Chr.19	Chr.05(A05)	191.237
139	NAU1042a	Chr.05--Chr.19	Chr.05(A05)	51.594
140	NAU1042b	Chr.05--Chr.19	Chr.19(D05)	63.445
141	NAU1042c	Chr.05--Chr.19	Chr.05(A05)	58.648
142	NAU1221a	Chr.05--Chr.19	Chr.05(A05)	52.043
143	NAU1221b	Chr.05--Chr.19	Chr.19(D05)	62.861
144	NAU1221c	Chr.05--Chr.19	Chr.05(A05)	58.273
145	TMB0131a	Chr.05--Chr.19	Chr.19(D05)	97.587

146	TMB0131b	Chr.05--Chr.19	Chr.05(A05)	95.305
147	TMB0189a	Chr.05--Chr.19	Chr.05(A05)	62.021
148	TMB0189b	Chr.05--Chr.19	Chr.19(D05)	68.397
149	TMB0835a	Chr.05--Chr.19	Chr.05(A05)	12.165
150	TMB0835b	Chr.05--Chr.19	Chr.19(D05)	10.41
151	TMB0865a	Chr.05--Chr.19	Chr.19(D05)	95.403
152	TMB0865b	Chr.05--Chr.19	Chr.05(A05)	88.86
153	TMB1282a	Chr.05--Chr.19	Chr.05(A05)	94.087
154	TMB1282b	Chr.05--Chr.19	Chr.19(D05)	100.166
155	TMB1418a	Chr.05--Chr.19	Chr.19(D05)	9.974
156	TMB1418b	Chr.05--Chr.19	Chr.05(A05)	12.228
157	TMB1489a	Chr.05--Chr.19	Chr.19(D05)	96.268
158	TMB1489b	Chr.05--Chr.19	Chr.05(A05)	90.358
159	TMC005a	Chr.05--Chr.19	Chr.05(A05)	62.056
160	TMC005b	Chr.05--Chr.19	Chr.19(D05)	68.522
161	BNL0584a	Chr.06--Chr.25	Chr.06(A06)	123.038
162	BNL0584b	Chr.06--Chr.25	Chr.25(D06)	116.445
163	BNL0827a	Chr.06--Chr.25	Chr.06(A06)	122.626
164	BNL0827b	Chr.06--Chr.25	Chr.25(D06)	116.262
165	BNL1169a	Chr.06--Chr.25	Chr.25(D06)	55.072
166	BNL1169b	Chr.06--Chr.25	Chr.06(A06)	56.306
167	BNL1440a	Chr.06--Chr.25	Chr.25(D06)	65.308
168	BNL1440b	Chr.06--Chr.25	Chr.06(A06)	68.814
169	BNL3594a	Chr.06--Chr.25	Chr.06(A06)	12.266
170	BNL3594b	Chr.06--Chr.25	Chr.25(D06)	12.858
171	MUSS275a	Chr.06--Chr.25	Chr.06(A06)	55.735
172	MUSS275b	Chr.06--Chr.25	Chr.25(D06)	55.885
173	MUSS501a	Chr.06--Chr.25	Chr.06(A06)	54.455
174	MUSS501b	Chr.06--Chr.25	Chr.25(D06)	51.867
175	NAU0905a	Chr.06--Chr.25	Chr.06(A06)	50.834
176	NAU0905b	Chr.06--Chr.25	Chr.25(D06)	51.352
177	NAU2238a	Chr.06--Chr.25	Chr.06(A06)	55.752
178	NAU2238b	Chr.06--Chr.25	Chr.25(D06)	55.968
179	TMB0436a	Chr.06--Chr.25	Chr.25(D06)	56.048
180	TMB0436b	Chr.06--Chr.25	Chr.06(A06)	55.901
181	TMB1740b	Chr.06--Chr.25	Chr.25(D06)	2.324
182	TMB1740c	Chr.06--Chr.25	Chr.06(A06)	0
183	UCD311a	Chr.06--Chr.25	Chr.25(D06)	66.321
184	UCD311b	Chr.06--Chr.25	Chr.06(A06)	69.673
185	BNL0580a	Chr.07--Chr.16	Chr.07(A07)	64.876
186	BNL0580b	Chr.07--Chr.16	Chr.16(D07)	56.531
187	BNL1026a	Chr.07--Chr.16	Chr.07(A07)	86.925
188	BNL1026b	Chr.07--Chr.16	Chr.16(D07)	82.924
189	BNL1122a	Chr.07--Chr.16	Chr.16(D07)	69.501
190	BNL1122b	Chr.07--Chr.16	Chr.07(A07)	84.894
191	BNL1395b	Chr.07--Chr.16	Chr.16(D07)	80.877
192	BNL1395c	Chr.07--Chr.16	Chr.07(A07)	91.646
193	BNL1531a	Chr.07--Chr.16	Chr.16(D07)	37.904
194	BNL1531b	Chr.07--Chr.16	Chr.07(A07)	43.294
195	BNL1604a	Chr.07--Chr.16	Chr.07(A07)	93.221

196	BNL1604b	Chr.07--Chr.16	Chr.16(D07)	92.755
197	BNL1694a	Chr.07--Chr.16	Chr.16(D07)	78.221
198	BNL1694b	Chr.07--Chr.16	Chr.07(A07)	85.877
199	BNL3319a	Chr.07--Chr.16	Chr.16(D07)	70.988
200	BNL3319b	Chr.07--Chr.16	Chr.07(A07)	79.843
201	BNL3793a	Chr.07--Chr.16	Chr.07(A07)	44.239
202	BNL3793b	Chr.07--Chr.16	Chr.16(D07)	37.067
203	CM0066a	Chr.07--Chr.16	Chr.07(A07)	87.574
204	CM0066b	Chr.07--Chr.16	Chr.16(D07)	81.85
205	JESPR228a	Chr.07--Chr.16	Chr.07(A07)	86.059
206	JESPR228b	Chr.07--Chr.16	Chr.16(D07)	76.369
207	MGHES058a	Chr.07--Chr.16	Chr.07(A07)	32.195
208	MGHES058b	Chr.07--Chr.16	Chr.16(D07)	26.051
209	MUSB1181a	Chr.07--Chr.16	Chr.07(A07)	27.972
210	MUSB1181b	Chr.07--Chr.16	Chr.16(D07)	50.696
211	NAU2002a	Chr.07--Chr.16	Chr.07(A07)	53.706
212	NAU2002b	Chr.07--Chr.16	Chr.16(D07)	46.276
213	STV023a	Chr.07--Chr.16	Chr.07(A07)	70.789
214	STV023b	Chr.07--Chr.16	Chr.16(D07)	62.828
215	TMB0009a	Chr.07--Chr.16	Chr.16(D07)	84.219
216	TMB0009b	Chr.07--Chr.16	Chr.07(A07)	87.45
217	TMB0180a	Chr.07--Chr.16	Chr.16(D07)	84.367
218	TMB0180b	Chr.07--Chr.16	Chr.07(A07)	87.532
219	TMB2566a	Chr.07--Chr.16	Chr.07(A07)	92.08
220	TMB2566b	Chr.07--Chr.16	Chr.16(D07)	84.914
221	BNL0387a	Chr.08--Chr.24	Chr.24(D08)	30.31
222	BNL0387b	Chr.08--Chr.24	Chr.08(A08)	55.645
223	BNL2961a	Chr.08--Chr.24	Chr.24(D08)	61.118
224	BNL2961b	Chr.08--Chr.24	Chr.08(A08)	79.829
225	CIR278a	Chr.08--Chr.24	Chr.24(D08)	8.841
226	CIR278b	Chr.08--Chr.24	Chr.08(A08)	39.176
227	CM0043a	Chr.08--Chr.24	Chr.24(D08)	19.176
228	CM0043b	Chr.08--Chr.24	Chr.08(A08)	49.79
229	JESPR157a	Chr.08--Chr.24	Chr.08(A08)	115.376
230	JESPR157b	Chr.08--Chr.24	Chr.24(D08)	96.063
231	JESPR291a	Chr.08--Chr.24	Chr.24(D08)	52.732
232	JESPR291b	Chr.08--Chr.24	Chr.08(A08)	0
233	MUSS250a	Chr.08--Chr.24	Chr.24(D08)	73.494
234	MUSS250b	Chr.08--Chr.24	Chr.08(A08)	88.436
235	NAU1322b	Chr.08--Chr.24	Chr.24(D08)	28.003
236	NAU1322c	Chr.08--Chr.24	Chr.08(A08)	51.176
237	TMB1639a	Chr.08--Chr.24	Chr.08(A08)	68.111
238	TMB1639b	Chr.08--Chr.24	Chr.24(D08)	45.159
239	BNL0686a	Chr.09--Chr.23	Chr.23(D09)	141.353
240	BNL0686b	Chr.09--Chr.23	Chr.09(A09)	139.427
241	BNL1030a	Chr.09--Chr.23	Chr.09(A09)	52.347
242	BNL1030b	Chr.09--Chr.23	Chr.23(D09)	55.034
243	BNL1317a	Chr.09--Chr.23	Chr.09(A09)	63.911
244	BNL1317b	Chr.09--Chr.23	Chr.23(D09)	54.304
245	BNL1414a	Chr.09--Chr.23	Chr.09(A09)	51.93

246	BNL1414b	Chr.09--Chr.23	Chr.23(D09)	54.881
247	BNL1672a	Chr.09--Chr.23	Chr.09(A09)	91.507
248	BNL1672b	Chr.09--Chr.23	Chr.23(D09)	91.644
249	BNL2590a	Chr.09--Chr.23	Chr.23(D09)	45.018
250	BNL2590b	Chr.09--Chr.23	Chr.09(A09)	44.111
251	BNL3031a	Chr.09--Chr.23	Chr.09(A09)	90.519
252	BNL3031b	Chr.09--Chr.23	Chr.23(D09)	92.688
253	BNL3140a	Chr.09--Chr.23	Chr.23(D09)	69.094
254	BNL3140c	Chr.09--Chr.23	Chr.09(A09)	73.214
255	BNL3410a	Chr.09--Chr.23	Chr.09(A09)	80.672
256	BNL3410b	Chr.09--Chr.23	Chr.23(D09)	82.559
257	CIR019a	Chr.09--Chr.23	Chr.09(A09)	121.497
258	CIR019b	Chr.09--Chr.23	Chr.23(D09)	115.255
259	CIR353a	Chr.09--Chr.23	Chr.23(D09)	107.484
260	CIR353b	Chr.09--Chr.23	Chr.09(A09)	115.047
261	CM0007a	Chr.09--Chr.23	Chr.09(A09)	51.656
262	CM0007b	Chr.09--Chr.23	Chr.23(D09)	55.351
263	CM0071a	Chr.09--Chr.23	Chr.09(A09)	49.248
264	CM0071b	Chr.09--Chr.23	Chr.23(D09)	54.871
265	HAU045a	Chr.09--Chr.23	Chr.09(A09)	105.855
266	HAU045b	Chr.09--Chr.23	Chr.23(D09)	102.291
267	JESPR095a	Chr.09--Chr.23	Chr.09(A09)	15.424
268	JESPR095b	Chr.09--Chr.23	Chr.23(D09)	18.39
269	JESPR208a	Chr.09--Chr.23	Chr.09(A09)	51.66
270	JESPR208b	Chr.09--Chr.23	Chr.23(D09)	54.883
271	JESPR247a	Chr.09--Chr.23	Chr.09(A09)	114.465
272	JESPR247b	Chr.09--Chr.23	Chr.23(D09)	104.781
273	JESPR248a	Chr.09--Chr.23	Chr.09(A09)	66.239
274	JESPR248b	Chr.09--Chr.23	Chr.23(D09)	66.478
275	JESPR274a	Chr.09--Chr.23	Chr.09(A09)	114.328
276	JESPR274b	Chr.09--Chr.23	Chr.23(D09)	104.97
277	MGHES002a	Chr.09--Chr.23	Chr.23(D09)	52.056
278	MGHES002b	Chr.09--Chr.23	Chr.09(A09)	50.396
279	MGHES046a	Chr.09--Chr.23	Chr.09(A09)	41.225
280	MGHES046b	Chr.09--Chr.23	Chr.23(D09)	42.385
281	MUCS080a	Chr.09--Chr.23	Chr.09(A09)	69.916
282	MUCS080b	Chr.09--Chr.23	Chr.23(D09)	67.028
283	MUSS083a	Chr.09--Chr.23	Chr.09(A09)	45.479
284	MUSS083b	Chr.09--Chr.23	Chr.23(D09)	49.208
285	MUSS298a	Chr.09--Chr.23	Chr.23(D09)	26.144
286	MUSS298c	Chr.09--Chr.23	Chr.09(A09)	23.992
287	NAU2200a	Chr.09--Chr.23	Chr.09(A09)	106.094
288	NAU2200b	Chr.09--Chr.23	Chr.23(D09)	102.296
289	TMB0670a	Chr.09--Chr.23	Chr.23(D09)	123.938
290	TMB0670b	Chr.09--Chr.23	Chr.09(A09)	128.15
291	UCD248a	Chr.09--Chr.23	Chr.23(D09)	58.512
292	UCD248b	Chr.09--Chr.23	Chr.09(A09)	57.989
293	BNL3071a	Chr.10--Chr.20	Chr.20(D10)	73.613
294	BNL3071b	Chr.10--Chr.20	Chr.10(A10)	53.133
295	DPL0317a	Chr.10--Chr.20	Chr.20(D10)	7.72

296	DPL0317b	Chr.10--Chr.20	Chr.10(A10)	51.45
297	JESPR006a	Chr.10--Chr.20	Chr.10(A10)	63.34
298	JESPR006e	Chr.10--Chr.20	Chr.20(D10)	56.843
299	MUSS096a	Chr.10--Chr.20	Chr.10(A10)	17.905
300	MUSS096b	Chr.10--Chr.20	Chr.20(D10)	88.794
301	MUSS135a	Chr.10--Chr.20	Chr.20(D10)	39.71
302	MUSS135b	Chr.10--Chr.20	Chr.10(A10)	101.936
303	MUSS347a	Chr.10--Chr.20	Chr.20(D10)	70.713
304	MUSS347b	Chr.10--Chr.20	Chr.10(A10)	53.911
305	NAU2139a	Chr.10--Chr.20	Chr.20(D10)	119.011
306	NAU2139b	Chr.10--Chr.20	Chr.10(A10)	13.801
307	TMB0161a	Chr.10--Chr.20	Chr.20(D10)	76.434
308	TMB0161b	Chr.10--Chr.20	Chr.10(A10)	42.366
309	TMB0812a	Chr.10--Chr.20	Chr.20(D10)	59.277
310	TMB0812b	Chr.10--Chr.20	Chr.10(A10)	69.641
311	TMB0858a	Chr.10--Chr.20	Chr.20(D10)	59.645
312	TMB0858b	Chr.10--Chr.20	Chr.10(A10)	69.852
313	BNL1034a	Chr.11--Chr.21	Chr.21(D11)	43.268
314	BNL1034b	Chr.11--Chr.21	Chr.11(A11)	31.807
315	BNL1231a	Chr.11--Chr.21	Chr.21(D11)	113.359
316	BNL1231b	Chr.11--Chr.21	Chr.11(A11)	140.859
317	BNL2895a	Chr.11--Chr.21	Chr.21(D11)	91.359
318	BNL2895b	Chr.11--Chr.21	Chr.11(A11)	104.351
319	BNL4011a	Chr.11--Chr.21	Chr.21(D11)	109.304
320	BNL4011c	Chr.11--Chr.21	Chr.11(A11)	136.826
321	CIR051a	Chr.11--Chr.21	Chr.11(A11)	43.969
322	CIR051b	Chr.11--Chr.21	Chr.21(D11)	53.635
323	CIR196a	Chr.11--Chr.21	Chr.11(A11)	145.826
324	CIR196b	Chr.11--Chr.21	Chr.21(D11)	120.718
325	CIR316a	Chr.11--Chr.21	Chr.11(A11)	155.978
326	CIR316b	Chr.11--Chr.21	Chr.21(D11)	128
327	DPL0528a	Chr.11--Chr.21	Chr.21(D11)	80.952
328	DPL0528b	Chr.11--Chr.21	Chr.11(A11)	82.39
329	JESPR211a	Chr.11--Chr.21	Chr.21(D11)	95.642
330	JESPR211c	Chr.11--Chr.21	Chr.21(D11)	103.858
331	JESPR244a	Chr.11--Chr.21	Chr.21(D11)	96.81
332	JESPR244b	Chr.11--Chr.21	Chr.21(D11)	105.694
333	JESPR245a	Chr.11--Chr.21	Chr.21(D11)	86.302
334	JESPR245b	Chr.11--Chr.21	Chr.11(A11)	89.968
335	MGHES016a	Chr.11--Chr.21	Chr.11(A11)	146.45
336	MGHES016b	Chr.11--Chr.21	Chr.21(D11)	119.469
337	MUCS088a	Chr.11--Chr.21	Chr.11(A11)	144.955
338	MUCS088b	Chr.11--Chr.21	Chr.21(D11)	122.637
339	MUSB0641b	Chr.11--Chr.21	Chr.11(A11)	107.855
340	MUSB0641f	Chr.11--Chr.21	Chr.21(D11)	85.65
341	MUSB0849a	Chr.11--Chr.21	Chr.21(D11)	87.285
342	MUSB0849b	Chr.11--Chr.21	Chr.11(A11)	96.499
343	NAU2016a	Chr.11--Chr.21	Chr.11(A11)	146.951
344	NAU2016b	Chr.11--Chr.21	Chr.21(D11)	120.702
345	NAU2152a	Chr.11--Chr.21	Chr.11(A11)	141.606

346	NAU2152b	Chr.11--Chr.21	Chr.21(D11)	116.093
347	STV069a	Chr.11--Chr.21	Chr.11(A11)	30.503
348	STV069b	Chr.11--Chr.21	Chr.21(D11)	42.017
349	TMA012a	Chr.11--Chr.21	Chr.21(D11)	59.891
350	TMA012b	Chr.11--Chr.21	Chr.11(A11)	47.664
351	TMB0043a	Chr.11--Chr.21	Chr.21(D11)	59.715
352	TMB0043b	Chr.11--Chr.21	Chr.11(A11)	48.088
353	TMB0426b	Chr.11--Chr.21	Chr.21(D11)	91.997
354	TMB0426c	Chr.11--Chr.21	Chr.11(A11)	105.587
355	TMB0628a	Chr.11--Chr.21	Chr.21(D11)	92.012
356	TMB0628b	Chr.11--Chr.21	Chr.11(A11)	105.382
357	TMB1637a	Chr.11--Chr.21	Chr.11(A11)	100.303
358	TMB1637b	Chr.11--Chr.21	Chr.21(D11)	94.595
359	UCD221a	Chr.11--Chr.21	Chr.21(D11)	75.84
360	UCD221b	Chr.11--Chr.21	Chr.11(A11)	66.613
361	BNL1227a	Chr.12--Chr.26	Chr.12(A12)	47.388
362	BNL1227b	Chr.12--Chr.26	Chr.26(D12)	32.151
363	BNL2621a	Chr.12--Chr.26	Chr.12(A12)	38.704
364	BNL2621c	Chr.12--Chr.26	Chr.26(D12)	18.905
365	BNL3537a	Chr.12--Chr.26	Chr.12(A12)	103.652
366	BNL3537b	Chr.12--Chr.26	Chr.26(D12)	80.605
367	BNL3599a	Chr.12--Chr.26	Chr.12(A12)	46.609
368	BNL3599b	Chr.12--Chr.26	Chr.26(D12)	25.875
369	BNL3867a	Chr.12--Chr.26	Chr.12(A12)	48.462
370	BNL3867b	Chr.12--Chr.26	Chr.26(D12)	28.288
371	CM0050a	Chr.12--Chr.26	Chr.12(A12)	42.186
372	CM0050b	Chr.12--Chr.26	Chr.26(D12)	24.645
373	DPL0039a	Chr.12--Chr.26	Chr.12(A12)	42.437
374	DPL0039b	Chr.12--Chr.26	Chr.26(D12)	24.225
375	GH243a	Chr.12--Chr.26	Chr.12(A12)	71.047
376	GH243b	Chr.12--Chr.26	Chr.26(D12)	50.405
377	JESPR121a	Chr.12--Chr.26	Chr.26(D12)	72.166
378	JESPR121b	Chr.12--Chr.26	Chr.12(A12)	93.015
379	JESPR295a	Chr.12--Chr.26	Chr.12(A12)	28.067
380	JESPR295b	Chr.12--Chr.26	Chr.26(D12)	9.226
381	JESPR300a	Chr.12--Chr.26	Chr.12(A12)	120.308
382	JESPR300b	Chr.12--Chr.26	Chr.26(D12)	109.912
383	MUSS101a	Chr.12--Chr.26	Chr.26(D12)	41.569
384	MUSS101b	Chr.12--Chr.26	Chr.12(A12)	60.075
385	NAU2251a	Chr.12--Chr.26	Chr.26(D12)	90.266
386	NAU2251b	Chr.12--Chr.26	Chr.12(A12)	108.675
387	BNL0243a	Chr.13--Chr.18	Chr.13(A13)	20.57
388	BNL0243b	Chr.13--Chr.18	Chr.18(D13)	6.145
389	BNL0569a	Chr.13--Chr.18	Chr.18(D13)	60.359
390	BNL0569b	Chr.13--Chr.18	Chr.13(A13)	88.631
391	BNL2667a	Chr.13--Chr.18	Chr.18(D13)	68.074
392	BNL2667b	Chr.13--Chr.18	Chr.13(A13)	97.746
393	BNL4029a	Chr.13--Chr.18	Chr.13(A13)	76.417
394	BNL4029b	Chr.13--Chr.18	Chr.18(D13)	47.973
395	CIR012a	Chr.13--Chr.18	Chr.18(D13)	47.444

396	CIR012b	Chr.13--Chr.18	Chr.13(A13)	74.583
397	CIR020a	Chr.13--Chr.18	Chr.13(A13)	60.846
398	CIR020b	Chr.13--Chr.18	Chr.18(D13)	35.94
399	GH678a	Chr.13--Chr.18	Chr.18(D13)	37.047
400	GH678b	Chr.13--Chr.18	Chr.13(A13)	62.974
401	JESPR016a	Chr.13--Chr.18	Chr.18(D13)	46.911
402	JESPR016b	Chr.13--Chr.18	Chr.13(A13)	75.405
403	JESPR153a	Chr.13--Chr.18	Chr.18(D13)	47.003
404	JESPR153b	Chr.13--Chr.18	Chr.13(A13)	74.995
405	MUCS145a	Chr.13--Chr.18	Chr.18(D13)	73.411
406	MUCS145b	Chr.13--Chr.18	Chr.13(A13)	100.402
407	MUSB0685a	Chr.13--Chr.18	Chr.18(D13)	54.301
408	MUSB0685c	Chr.13--Chr.18	Chr.13(A13)	84.045
409	GH252a	Chr.05--Chr.22	Chr.05(A05)	136.456
410	GH252b	Chr.05--Chr.22	Chr.22(D04)	18.384
411	BNL1044a	Chr.04--Chr.05	Chr.05(A05)	33.613
412	BNL1044c	Chr.04--Chr.05	Chr.04(A04)	48.104
413	TMB1025a	Chr.02--Chr.03	Chr.03(A03)	38.34
414	TMB1025b	Chr.02--Chr.03	Chr.02(A02)	55.406
415	BNL3545a	Chr.02--Chr.14	Chr.14(D03)	118.871
416	BNL3545c	Chr.02--Chr.14	Chr.02(A02)	117.493
417	JESPR156a	Chr.02--Chr.14	Chr.14(D03)	124.105
418	JESPR156b	Chr.02--Chr.14	Chr.02(A02)	112.254
419	NAU0895a	Chr.02--Chr.14	Chr.02(A02)	3.666
420	NAU0895b	Chr.02--Chr.14	Chr.14(D03)	0
421	NAU2265a	Chr.02--Chr.14	Chr.02(A02)	3.666
422	NAU2265b	Chr.02--Chr.14	Chr.14(D03)	0.324
423	BNL3408a	Chr.03--Chr.17	Chr.03(A03)	94.677
424	BNL3408b	Chr.03--Chr.17	Chr.17(D02)	14.258
425	CIR347a	Chr.03--Chr.17	Chr.17(D02)	13.884
426	CIR347b	Chr.03--Chr.17	Chr.03(A03)	95.503
427	MUSB1251a	Chr.03--Chr.17	Chr.17(D02)	66.379
428	MUSB1251c	Chr.03--Chr.17	Chr.17(D02)	49.98
429	TMB2069a	Chr.03--Chr.17	Chr.03(A03)	79.615
430	TMB2069b	Chr.03--Chr.17	Chr.17(D02)	38.045
431	UCD195a	Chr.03--Chr.17	Chr.17(D02)	35.996
432	UCD195b	Chr.03--Chr.17	Chr.03(A03)	85.084
433	UCD277a	Chr.03--Chr.17	Chr.17(D02)	36.269
434	UCD277b	Chr.03--Chr.17	Chr.03(A03)	85.201
435	CM0160a	Intrachromosomal	Chr.21(D11)	95.408
436	CM0160c	Intrachromosomal	Chr.21(D11)	103.898
437	JESPR211a	Intrachromosomal	Chr.21(D11)	91.764
438	JESPR211c	Intrachromosomal	Chr.21(D11)	109.903
439	JESPR244a	Intrachromosomal	Chr.21(D11)	92.558
440	JESPR244b	Intrachromosomal	Chr.21(D11)	111.955
441	TMB0426a	Intrachromosomal	Chr.11(A11)	116.728
442	TMB0426c	Intrachromosomal	Chr.11(A11)	109.331
443	BNL1161a	Non-homeologous	Chr.10(A10)	57.555
444	BNL1161b	Non-homeologous	Chr.23(D09)	125.676
445	BNL1669a	Non-homeologous	Chr.10(A10)	59.419

446	BNL1669b	Non-homeologous	Chr.26(D12)	33.43
447	BNL3280a	Non-homeologous	Chr.20(D10)	103.417
448	BNL3280b	Non-homeologous	Chr.18(D13)	47.32
449	BNL3886a	Non-homeologous	Chr.12(A12)	57.27
450	BNL3886b	Non-homeologous	Chr.01(A01)	68.641
451	CIR372a	Non-homeologous	Chr.09(A09)	118.866
452	CIR372b	Non-homeologous	Chr.10(A10)	97.831
453	GH681a	Non-homeologous	Chr.03(A03)	55.211
454	GH681b	Non-homeologous	Chr.13(A13)	39.19
455	JESPR007a	Non-homeologous	Chr.18(D13)	37.887
456	JESPR007b	Non-homeologous	Chr.20(D10)	64.419
457	JESPR227a	Non-homeologous	Chr.02(A02)	67.265
458	JESPR227b	Non-homeologous	Chr.25(D06)	67.201
459	MUSB0625b	Non-homeologous	Chr.07(A07)	66.459
460	MUSB0625f	Non-homeologous	Chr.10(A10)	92.708
461	MUSB0812a	Non-homeologous	Chr.16(D07)	30.806
462	MUSB0812c	Non-homeologous	Chr.08(A08)	137.247
463	MUSB1064a	Non-homeologous	Chr.10(A10)	62.178
464	MUSB1064d	Non-homeologous	Chr.01(A01)	37.027
465	TMB0694a	Non-homeologous	Chr.07(A07)	87.414
466	TMB0694b	Non-homeologous	Chr.15(D01)	39.88
467	TMB1745a	Non-homeologous	Chr.10(A10)	77.59
468	TMB1745b	Non-homeologous	Chr.24(D08)	14.575
469	TMB1809a	Non-homeologous	Chr.08(A08)	99.711
470	TMB1809b	Non-homeologous	Chr.18(D13)	47.173
471	BNL2634a	unknown	Unmapped	
472	BNL2634b	unknown	Chr.16(D07)	41.553
473	GH247a	unknown	Unmapped	
474	GH247b	unknown	Chr.09(A09)	61.823
475	GH310a	unknown	Unmapped	
476	GH310b	unknown	Chr.09(A09)	68.142
477	GH350a	unknown	Chr.15(D01)	48.962
478	GH350b	unknown	Unmapped	
479	MUSB0139b	unknown	Unmapped	
480	MUSB0139c	unknown	Chr.07(A07)	102.315
481	MUSB0369a	unknown	Unmapped	
482	MUSB0369b	unknown	Chr.11(A11)	103.532
483	MUSS009a	unknown	Unmapped	
484	MUSS009b	unknown	Unmapped	
485	MUSS049a	unknown	Unmapped	
486	MUSS049b	unknown	Unmapped	
487	STV031a	unknown	Unmapped	
488	STV031b	unknown	Chr.20(D10)	9.99
489	TMB1483a	unknown	Unmapped	
490	TMB1483b	unknown	Chr.06(A06)	75.663
491	TMB1888b	unknown	Chr.12(A12)	23.447
492	TMB1888c	unknown	Unmapped	
493	TMB2295a	unknown	Unmapped	
494	TMB2295b	unknown	Chr.18(D13)	104.835
