# Supplementary material for: A High-Density Simple Sequence Repeat and Single Nucleotide Polymorphism Genetic Map of the Tetraploid Cotton Genome
Source: G3 (Bethesda). 2012 Jan 1;2(1):43–58. doi: 10.1534/g3.111.001552 (PMC3276184; doi:10.1534/g3.111.001552)
Supplement: Supporting Information [file supp_2.1.43_TableS1.pdf]

**Table S1 Distribution of 247 SNP and 310 TMB markers among the 26 chromosomes.**

| Chromosome | # Markers | | Chromosome | # Markers | |
|---|---|---|---|---|---|
| | SNP | TMB | | SNP | TMB |
| Chr.01(A01) | 4 | 12 | Chr.15(D01) | 4 | 17 |
| Chr.02(A02) | 10 | 9 | Chr.17(D02) | 1 | 8 |
| Chr.03(A03) | 12 | 12 | Chr.14(D03) | 2 | 16 |
| Chr.04(A04) | 16 | 4 | Chr.22(D04) | 2 | 3 |
| Chr.05(A05) | 35 | 17 | Chr.19(D05) | 7 | 22 |
| Chr.06(A06) | 10 | 18 | Chr.25(D06) | 1 | 12 |
| Chr.07(A07) | 19 | 12 | Chr.16(D07) | 1 | 8 |
| Chr.08(A08) | 20 | 12 | Chr.24(D08) | 2 | 10 |
| Chr.09(A09) | 10 | 9 | Chr.23(D09) | 5 | 16 |
| Chr.10(A10) | 19 | 11 | Chr.20(D10) | 4 | 17 |
| Chr.11(A11) | 36 | 16 | Chr.21(D11) | 3 | 18 |
| Chr.12(A12) | 13 | 8 | Chr.26(D12) | 3 | 2 |
| Chr.13(A13) | 7 | 11 | Chr.18(D13) | 1 | 10 |
| Subtotal-At | 211 | 151 | Subtotal-Dt | 36 | 159 |
