# Supplementary material for: A High-Density Simple Sequence Repeat and Single Nucleotide Polymorphism Genetic Map of the Tetraploid Cotton Genome
Source: G3 (Bethesda). 2012 Jan 1;2(1):43–58. doi: 10.1534/g3.111.001552 (PMC3276184; doi:10.1534/g3.111.001552)
Supplement: Supporting Information [file supp_2.1.43_TableS2.pdf]

Table S2   SSR and SNP marker loci that are either Identical or co-segregated in the TM-1 x 3-79 RIL mapping population.

#	Marker locus 1 identical to	Marker locus 2
1	BNL3545a	BNL3545b
2	DPL0031a	UCcg10265_529
3	DPL0215	UCcot10558_399
4	DPL0264	DPL0854
5	DPL0301	TMB0436a
6	JESPR215	JESPR229
7	NAU0934b	NAU0980
8	UCcg10010_234	UCcg10010_475
9	UCcg10295_217	UCcot10031_405
10	UCcg10310_614	UCcg10809_201
11	UCcg10436_1035	UCcg10436_1135
12	UCcg10436_1035	UCcg10436_861
13	UCcg10503_331	UCcg10503_86
14	UCcg10614_274	UCcg10614_520
15	UCcg10682_477	UCcg10682_712
16	UCcg10770_168	UCcg10770_48
17	UCcg10773_103	UCcg10773_312
18	UCcg10797_372	UCcg10797_669
19	UCcg10816_112	UCcg10816_726
20	UCcg10855_353	UCcg10855_642
21	UCcg10883_389	UCcg10883_495
22	UCcg10998_153	UCcg10998_265
23	UCcg11002_222	UCcg11002_302
24	UCcg11002_222	UCcot10019_389
25	UCcg11015_199	UCcg11015_757
26	UCcg11073_51	UCcg11073_768
27	UCcg11135_141	UCcg11135_438
28	UCcg11256_182	UCcg11256_354
29	UCcg11440_119	UCcg11440_818
30	UCcgs10034_277	UCcgs10034_82
31	UCcot10030_1469	UCcot10030_820
32	UCcot10031_1194	UCcot10112_120
33	UCcot10031_1194	UCcot10112_228

34	UCcot10055_499	UCcot10055_591
35	UCcot10112_120	UCcot10112_228
36	UCcot10128_122	UCcot10128_269
37	UCcot10138_116	UCcot10138_775
38	UCcot10157_256	UCcot10157_97
39	UCcot10162_137	UCcot10162_64
40	UCcot10213_226	UCcot10213_61
41	UCcot10242_135	UCcot10242_76
42	UCcot10318_484	UCcot10318_588
43	UCcot10393_208	UCcot10393_558
44	UCcot10397_424	UCcot10397_643
45	UCcot10434_204	UCcot10434_536
46	UCcot10465_113	UCcot10465_333
47	UCcot10493_151	UCcot10493_618
